# High-throughput in situ sizing and quantum yield determination of individual perovskite nanocrystals

**DOI:** 10.1038/s41563-026-02607-5

**Published:** 2026-05-21

**Authors:** Christoph G. Gruber, Andrea Mancini, Nina A. Henke, Carola Lampe, Olivier Henrotte, Michael F. Lichtenegger, Franz Gröbmeyer, Andreas Singldinger, Yi Li, Stefan A. Maier, Alexander S. Urban, Emiliano Cortés

**Affiliations:** 1 Nanoinstitute Munich, Faculty of Physics, Ludwig-Maximilians-Universität München, Munich, Germany; 2 Nanospectroscopy Group, Nanoinstitute Munich, Faculty of Physics, Ludwig-Maximilians-Universität München, Munich, Germany; 3 School of Microelectronics, MOE Engineering Research Center of Integrated Circuits for Next Generation Communications, Southern University of Science and Technology, Shenzhen, China; 4 School of Physics and Astronomy, Monash University, Clayton, Victoria Australia; 5 Department of Physics, Imperial College London, London, UK

**Keywords:** Quantum dots, Design, synthesis and processing, Electronic devices

## Abstract

Colloidal nanocrystals exhibit high tunability and low-cost solution processing attractive for next-generation electronic applications. However, colloidal nanocrystals are inherently heterogeneous and the impact of this heterogeneity on device performance has been largely disregarded, since analytical techniques cannot assess the functionality of individual nanocrystals on a large scale. Here we introduce a rapid, in situ method to determine the size and quantum yield of thousands of individual nanocrystals within minutes, based on interferometric scattering microscopy and photoluminescence imaging. Monitoring the life cycle of CsPbBr_3_ perovskite nanocubes, our approach uncovers phenomena masked by bulk averaging. We find a substantial performance spread across the nanocubes and an anticorrelation of quantum yield and size. During a subsequent solution-phase defect engineering process, we uncover size-dependent enhancement kinetics, which initially favour the enhancement of smaller nanocubes. Finally, we image light-induced degradation by tracking the size reduction and photobleaching of single sub-20-nm nanocrystals, finding material loss decreases at higher laser powers due to the trapping of photoinduced electrons by the formed metallic lead.

## Abstract (web summary)

Interferometric scattering microscopy combined with photoluminescence imaging enables the fast, high-throughput screening of size and quantum yield for thousands of perovskite nanocubes at the single-particle level across their entire life cycle.

## Main

Semiconductor nanocrystals, particularly comprising halide perovskites, can enable low-cost and large-area electronic devices through solution processing^1,2^. However, a major challenge in designing nanocrystal-based LEDs^3^, solar cells^4^ or single-photon emitters^5^ lies in the heterogeneity of colloidal samples. These samples contain billions of individual nanoparticles with varying properties^6,7^. Consequently, the stability, efficiency and fabrication of such devices rely on the precise control of the photophysical properties of the individual building blocks, that is, the luminescent nanocrystals. To achieve this control through either synthesis or post-synthesis routes, nanocrystal structure and functionality must be linked, which can ultimately be done through single-particle measurements. This remains challenging^6^.

On one hand, characterizing colloidal samples using single-particle data must rely on statistically meaningful quantities, with existing methods often being limited to a few particles^8,9^. Moreover, for luminescent nanocrystals, the key parameters that determine their functionality, namely, particle size and photoluminescence quantum yield (PLQY), must be retrieved in situ. This is particularly complex for the PLQY ($\frac{\mathrm{Emitted}\,\mathrm{photons}}{\mathrm{Absorbed}\,\mathrm{photons}}\,$) due to the difficulty in determining the number of absorbed photons, given the stark mismatch between the absorption cross-section and a diffraction-limited focal spot. Although the lack of techniques to obtain size and PLQY single-particle data has been acknowledged as a major challenge^6^, and various efforts have been made, such as using electron microscopy or atomic force microscopy^10–13^, realization in a solution environment and at statistically meaningful quantities has yet to be achieved. Therefore, common characterization remains sample averaged, involving size distributions via ex situ transmission electron microscopy (TEM) analysis and ensemble PLQY measurements^14^. This approach, however, misses the immense potential in identifying the functional heterogeneity inside colloidal samples, which is the foundation for the rational selection and improvement of nanocrystal components.

Here we establish a method based on interferometric scattering (iSCAT)^15,16^ and photoluminescence (PL) microscopy to directly correlate size, emission and PLQY of thousands of individual perovskite nanocrystals in situ. iSCAT is a method capable of determining the size of single nanoparticles in solution and provides label-free and universal sensitivity by relying on light scattering, a property inherent to all matter^17–19^. It can achieve sensitivity below five nanometres at high speeds (microseconds/milliseconds) and can be coupled with other optical detection channels such as fluorescence microscopy^20–23^. This technique, under the name of mass photometry, has found great success in biology for its quantitative use in determining the volume and, therefore, the molecular mass of biological macromolecules such as proteins^24,25^. In our study, we combine the quantitative aspects of iSCAT and mass photometry with quantitative emission measurements for semiconductor nanocrystals, allowing us to determine the PLQY of single particles in high throughput. We focus on colloidal CsPbBr_3_ perovskite nanocubes, one of the most studied nanocrystals due to their excellent optical properties. They come, however, with substantial challenges, such as a strong vulnerability to degradation, especially under operating conditions, and sample variability, with factors such as the number of defects, ligand concentration and nanocube concentration varying from batch to batch as well as in time^14^. We provide an insight into this key nanomaterial throughout its life cycle, starting with the as-synthesized colloidal solution, investigating performance enhancement via solution-phase defect repair, and finally degradation and nanocrystal ‘death’ under intense light illumination.

## Determining perovskite nanocrystal size simultaneous to emission

**Fig. 1:**
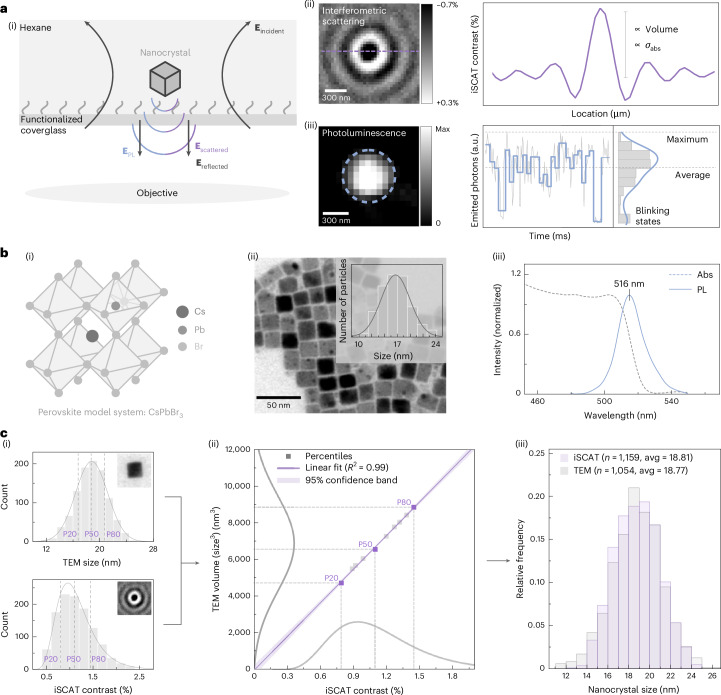
Determining the size of perovskite nanocrystals in colloidal solutions by iSCAT microscopy. **a**, Working principle of the all-optical method to determine particle size simultaneously to emission (i). On attaching to a functionalized coverglass, the PSF imaged via iSCAT microscopy contains the size information (ii). This is combined with PL microscopy to access the emission properties such as blinking as well as maximum and average emission of the attached particle (iii). Stable emission levels in the blinking traces are identified using CPA, yielding a noiseless model trace (blue line). **b**, CsPbBr_3_ perovskite nanocrystals. Sketch of the crystal structure (i); example TEM image of a colloidal sample (ii) and its corresponding size distribution (inset); bulk absorption (Abs) and emission properties (iii). **c**, Calibration method for converting measured iSCAT contrast to the nanocrystal size via TEM. Histograms of iSCAT contrast and TEM size distributions of the same colloidal CsPbBr_3_ sample (i). Percentiles of the distributions are indicated by dotted lines. Scatter plot of the percentiles (P20, P33, P40, P50, P60, P66, P75 and P80) of the iSCAT distribution and the TEM volume (*V* = size^3^) distribution (ii). The iSCAT contrast-to-volume conversion formula is obtained through the slope of a linear fit (${V}_{{\rm{cube}}}=6,082.7\,{\mathrm{nm}}^{3}\times {I}_{{\rm{iSCAT}}}$; shaded region, 95% confidence band of the fit), exploiting that the iSCAT contrast is proportional to the volume of the particle. Histograms of the iSCAT size distribution after applying the conversion formula and the TEM size distribution, showing high agreement (iii). **E**, electric field; *σ*_abs_, absorption cross-section.

**Extended Data Fig. 1:**
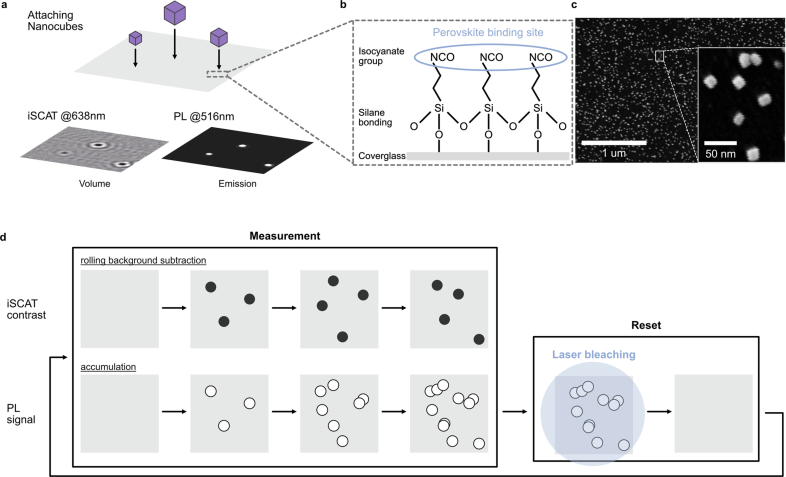
Sample design and measurement method. **a** In a typical measurement, nanocubes attach from solution and generate an optical readout (iSCAT excitation and detection at λ = 638 nm; PL excitation at λ = 450 nm; PL emission of the nanocubes around λ = 516 nm.) **b** Scheme of coverglass surface functionalization by a silane-agent (3-isocyanotopropyltrimethoxysilane). After the silane moiety forms a bond with the hydroxyl groups of the coverglass surface, the isocyanate functional group of the agent can function as a binding site for the solute perovskite cubes. **c** SEM image of the coverglass surface after one day in a sample holder filled with the typical nanocube solution. The image shows uniform surface coverage by single nanocubes that attached during this period. **d** Scheme of the methodology of the photobleaching-assisted high-throughput measurements. While the signals of attaching nanocubes in the iSCAT channel are continuously subtracted using the differential rolling average method, the emission signals in the PL channel accumulate and start to conceal the signals of newly attaching cubes. To reset the system to initial conditions with no detectable PL signal in the field of view, the measurement region is illuminated with a high power intensity before each measurement cycle to photobleach the emitting nanocubes that block the detection of new signals.

To enable the simultaneous optical determination of nanocrystal size and PL emission, we integrate two continuous-wave lasers into an inverted microscope, one above the bandgap (*λ* = 450 nm) to induce PL and one below the bandgap (*λ* = 638 nm), whose photons only interact via scattering with the nanocrystals (Fig. 1a). The colloidal CsPbBr_3_ nanocrystals used here possess the typical ABX_3_ perovskite structure (Fig. 1b(i))^26^, exhibit a size heterogeneity of 10–24 nm (equivalent to a volume of 1.000–13.800 nm^3^; Fig. 1b(ii) and Supplementary Fig. 1) and moderate colloidal stability enabled through the passivating oleylamine (OAm) and oleic acid (OAc) ligands^27^. The nanocrystals, being in the weakly quantum-confined regime, emit light at 516 nm (Fig. 1b(iii)). In a typical experiment, the nanocrystals (0.0014% of the initial 5-ml synthesis batch) are introduced into a hexane-filled sample holder, where they diffuse and attach to a functionalized coverglass (Extended Data Fig. 1a). We record both iSCAT signal (on attachment) and PL emission (of the attached nanocrystal), which allows us to determine the nanocrystal size, absorption cross-section and critical emission characteristics (Fig. 1a(ii),(iii)). The iSCAT signal, in the form of a point spread function (PSF) image, originates from the interference of light scattered by the nanocrystal with light reflected by the coverglass interface (Methods and Supplementary Section 2 provide the full methodology).

To determine a nanocrystal’s size through its iSCAT contrast (${I}_\mathrm{{iSCAT}}\,,$ the amplitude at the centre of the iSCAT PSF^24^), we calibrate the setup by correlating the size distribution of over 1,000 nanocubes from a single CsPbBr_3_ sample measured in a TEM device with the iSCAT contrast results obtained from the same sample (Fig. 1c(i)).

By calculating percentiles of the distributions, we obtain statistically relevant size and contrast values with the respective distributions encoded. After converting TEM size into volume (*V*_cube _= (nanocube size)^3^), we can linearly fit these data points to obtain the iSCAT contrast-to-volume conversion formula as ${I}_\mathrm{{iSCAT}}\propto {V}_\mathrm{{cube}}$ (Fig. 1c(ii); ${V}_{{\rm{cube}}}=6,082.7\,{\mathrm{nm}}^{3}\times {I}_{{\rm{iSCAT}}}$; *R* = 0.99). The resulting iSCAT-derived size distribution shows high agreement with the distribution obtained via TEM, confirming that iSCAT can be reliably used to determine the nanocube size (Fig. 1c(iii) and Supplementary Section 3b).

## Single-particle quantum yield for colloidal sample characterization

**Fig. 2:**
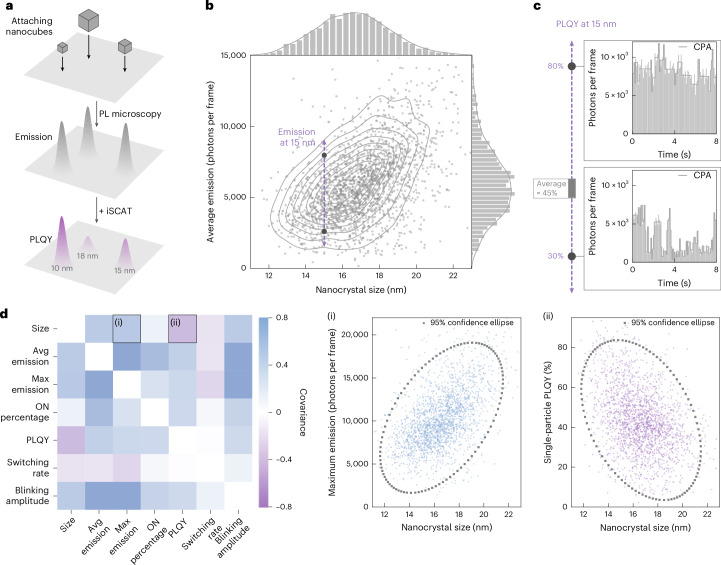
Simultaneous determination of particle size and emission unlocks single-particle quantum yield measurements and reveals a considerable, size-dependent spread in material quality inside the colloidal CsPbBr_3_ sample. **a**, Scheme showing that the emission values obtained by PL microscopy are insufficient to judge colloidal particles in terms of light conversion efficiency, that is, PLQY. Correlation with size is necessary to assess whether the light was, for example, emitted by a small particle with high efficiency or by a large particle with low efficiency. **b**, Scatter plot correlating emitted photons (PL) with particle size (iSCAT) for a colloidal CsPbBr_3_ sample (*n* = 2,179). The purple dashed line visualizes the spread of emission values for an example nanocube size of 15 nm, revealing a substantial variability in material performance. **c**, Experimental quantum yield and emission traces in time for two example 15-nm-sized nanocubes (indicated in **b**). The particle size determined by iSCAT microscopy enables the calculation of their absorption cross-section, consequently the number of absorbed photons and, ultimately, their PLQY. The quantum yield differs greatly compared with the sample-averaged value of 45% and the emission traces show considerable variations between the single particles in terms of blinking behaviour and absolute emission. **d**, Relationship between particle properties such as size, PLQY and key parameters extracted from the emission traces. The relationship is calculated and visualized by a covariance matrix. Here blue indicates a positive correlation, whereas purple indicates an inverse correlation. Scatter plot corresponding to the positive covariance value of nanocube size and maximum emission (i). The confidence interval visualizes the positive correlation, indicating that larger nanocubes emit more photons. Scatter plot corresponding to the negative covariance value of nanocube size and single-particle PLQY (ii). The confidence interval indicates the inverse relationship, meaning smaller sized nanocubes are surprisingly associated with higher PLQY values.

By establishing a nanoparticle size through the iSCAT contrast, we can now compute the particle’s absorption cross-section, the number of absorbed photons, and, combined with PL microscopy measurements of emitted photons, the PLQY (Fig. 2a and Methods). In general, the absorption cross-section depends in a non-trivial way not only on the particle size and material but also on its geometrical shape. However, for a deeply subwavelength particle, the absorption cross-section can be well approximated by the absorption of a small sphere^28^. Further, we assume a uniform dielectric function across the CsPbBr_3_ nanocrystal population (Methods and Supplementary Section 3d provide a detailed discussion on potential dielectric variability). We estimated a statistical error in determining the PLQY of around 10.4%, which is dominated by fluctuations associated with the measurement of the iSCAT contrasts (Supplementary Section 3c). To this end, we developed an automated, photobleaching-assisted method that combines iSCAT size information with quantitative PL measurements for large numbers of nanocrystals (>1,000 per hour; Extended Data Fig. 1d and Supplementary Section 4). We initially allow nanocrystals to attach, typically measuring up to 100 within a 2-min window. In iSCAT measurements, the signal of the attached nanocubes is continuously subtracted as background using a differential rolling average (DRA) method (Supplementary Section 2b), such that they are primarily resolved in the processed images on attachment^29,30^. By contrast, in PL microscopy, the emission signals accumulate over time and can obscure newly attaching nanocubes. To maintain a clear field of view, we periodically photobleach the attached nanocrystals by briefly increasing the excitation power. This resets the system and enables repeated measurement cycles of newly attaching nanocrystals (Extended Data Fig. 1d). After data acquisition, signals from the iSCAT and PL channels are automatically correlated (Supplementary Section 5). Applying this measurement method to over 2,000 CsPbBr_3_ nanocubes in hexane reveals a wide distribution of sizes and emitted photons, even among nanocubes of the same size (Fig. 2b (purple line) and Supplementary Video 1). This finding uncovers a substantial variability in material quality within the same colloidal sample, as illustrated by the emission and blinking characteristics of two 15 nm-sized nanocubes (Fig. 2c). As derived previously, we determined the PLQY for each nanocrystal in the ensemble (*n* > 2,000), revealing a sample-averaged PLQY value of 45% and a large spread for individual nanocubes (the two example cubes shown in Fig. 2c exhibited PLQY values of 30% and 80%). We attribute the large spread primarily to single-particle heterogeneity associated with non-radiative pathways originating from local disorder, particularly surface defects (Supplementary Section 3d). We validated the single-particle PLQY values obtained using our method by benchmarking them against two established bulk methods (integrating sphere and fluorescein dye reference), measured on the same day, from the same sample and at the same concentration (Supplementary Section 6). We extract blinking metrics using change point analysis (CPA; Methods)^31,32^, including switching rate (frequency of jumps with >20% of the maximum value), blinking amplitude and ON-state duration (≥70% of the maximum value). To provide a comprehensive overview and investigate correlations in this extensive dataset, we compute the covariance among the parameters (size, PLQY, emission and blinking metrics), which we show as a two-dimensional covariance map (Fig. 2d). Many of the parameters we track show a positive correlation, for example, the maximum and average emission with the nanocrystal size (Fig. 2d(i)). On the other hand, some parameters are essentially uncorrelated, such as the PLQY and the switching rate or the nanocrystal size with the ON percentage. In particular, we observe a strong anticorrelation between PLQY and nanocrystal size, that is, smaller nanocubes tend to have higher PLQY values (Fig. 2d(ii)). In general, PLQY can be governed by two contributions: changes in the radiative rate, for example, through size-dependent oscillator strength, or variations in the non-radiative rate associated with defect-mediated recombination. In the following, we combine our approach with in situ defect engineering to identify the mechanism responsible for the observed size dependence and to systematically probe the impact of different defect types on a single-particle basis.

## Single-particle quantum yield and size during solution-phase defect engineering

**Fig. 3:**
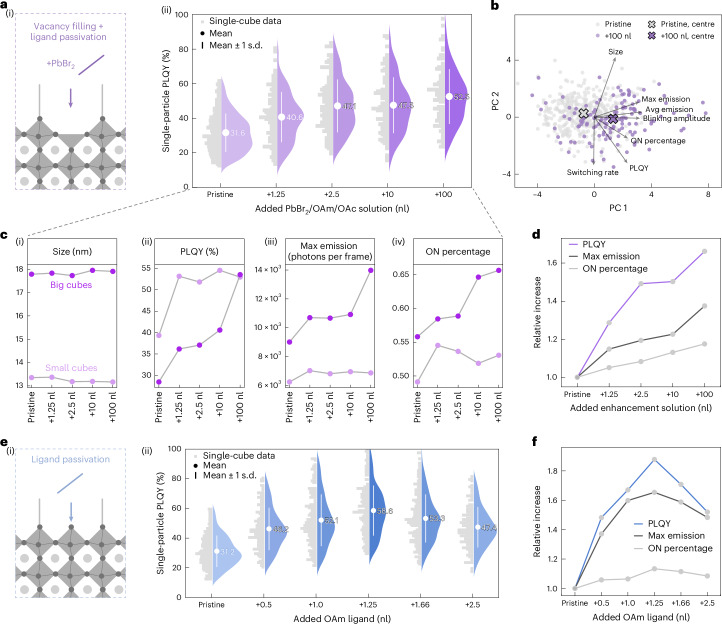
In situ single-particle quantum yield measurements during solution-phase defect repair reveal concealed effects on quantum yield populations, blinking behaviour and particle-size-dependent kinetics. **a**, In situ measurements of *n* = 907 colloidal CsPbBr_3_ nanocubes during post-synthesis treatment with a PbBr_2_, OAm and OAc mixture, termed enhancement solution. Scheme of the effect of enhancement solution on the nanocrystals—vacancy filling by PbBr_2_ salt and passivation of surface traps via ligand (purple line) binding (i). Single-particle quantum yield populations when adding the enhancement solution (ii). **b**, PCA of the dataset of **a** (for clarity, only pristine and +100-nl enhanced nanocubes are shown). A distinct clustering visualizes changes in the distribution of the two samples’ interdependent key parameters (visualized by arrows) in reduced dimensionality. **c**, Plots of key parameters during enhancement solution treatment (**b**) for two nanocube size subsets, namely, 12–14 nm (blue) and 17–19 nm (purple), reveal size-dependent kinetics (a guide to the eye is provided in grey). Each data point is the average of 30–90 individual cubes. The average nanocube size remains constant during the measurement, verifying our approach (i). Smaller nanocubes start with a higher absolute QY, get enhanced faster and then reach a plateau that larger nanocubes only reach after substantially more enhancement solution is added (ii). The trend of smaller nanocubes getting enhanced faster is also visible in their maximum emission and ON percentage; moreover, the enhancement solution affects ON percentage and maximum emission differently across addition steps (iii) and (iv). **d**, Relative increase in PLQY, ON percentage and maximum emission (over the entire nanocube population). The single-particle data show that a substantial part of the PLQY enhancement is due to reduced blinking. **e**, In situ measurements of *n* = 1,291 colloidal CsPbBr_3_ nanocubes during post-synthesis treatment with a solution of OAm. Scheme of the effect of OAm on the nanocrystals—passivation of surface traps via ligand (blue line) binding (i). Single-particle quantum yield populations when adding OAm ligand solution (ii). **f**, Relative increase in PLQY, ON percentage and maximum emission (over the entire nanocube population). The single-particle-based data show that the increase is primarily due to enhanced maximum emission.

**Extended Data Fig. 2:**
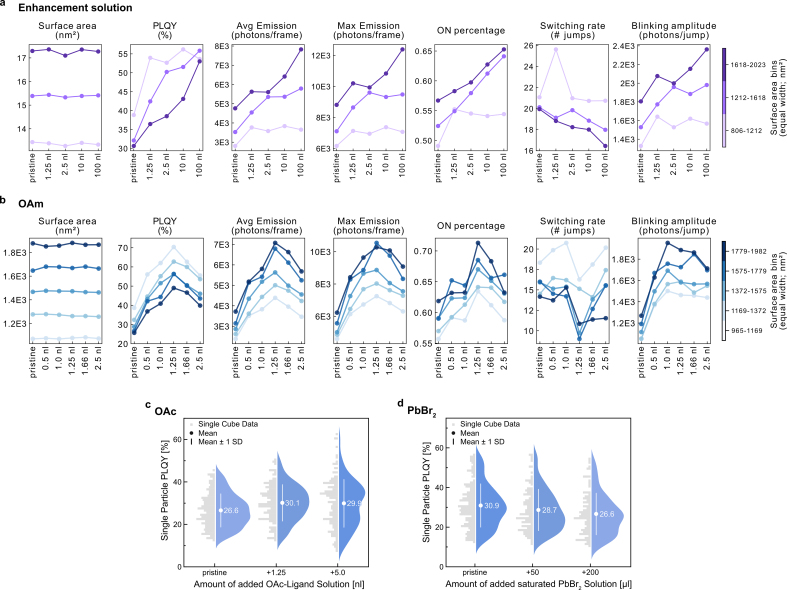
Effect of addition of OAc and PbBr_2_ on the nanocube population and surface area-dependent effects during addition of enhancement solution and OAm. **a** Enhancement solution. Evolution of key parameters during enhancement solution addition (Fig. 3a ii), grouped by nanocrystal surface area (SA). The crystal populations exhibit distinct trends: larger crystals start with lower PLQY values and show a delayed enhancement but eventually reach the PLQY levels of smaller cubes once a sufficient amount of enhancement solution has been added. **b** OAm ligand. Surface area-dependent kinetics of key parameters during addition of OAm ligand solution (Fig. 3e) grouped into subsets of surface area. The crystal populations follow the same trends, while smaller crystals retain higher PLQY levels throughout the additions, including at full coverage (1.25 nL). **c** OAc ligand. Evolution of in situ single particle PLQY distributions during addition of OAc ligand solution (n = 531). Alongside a slight increase in the average PLQY, the distributions exhibit changes in their spread. **d** Saturated PbBr_2_ solution (4 ppm in hexane; determined by atomic emission spectroscopy). Evolution of in situ single particle PLQY distributions during addition of PbBr_2_ salt solution (n = 457). The average PLQY decreases slightly with the amount added.

Numerous solution-phase defect-repair strategies exist to improve the post-synthesis material performance^14,33,34^. We focus on a widely used approach, enabling us to probe two types of defect: addition of lead(II) bromide (PbBr_2_) salt and the ligand pair OAm/OAc, referred to as the enhancement solution^35^. The salt mainly repairs surface halide vacancies, whereas the ligands (predominantly protonated OAm) bind to unpassivated surface ions, collectively boosting nanocrystal performance (Fig. 3a(i)). We gradually added the enhancement solution to the same diluted sample, monitoring >100 nanocrystals at each step. We find that the sample-averaged PLQY increased from 31.2% to 52.4% (~70% improvement) during the four addition steps (Fig. 3a(ii)). This trend is consistent with the expected defect-repair process and was corroborated by bulk measurements (Supplementary Section 7; variations in absolute values are due to differences in samples). To explore trends underlying the known ensemble properties of the nanocrystal solution, we analysed data from more than 900 individual nanocrystals. We started by applying principal component analysis (PCA) to simplify the complexity of our data and observe distinct clustering, pointing to notable changes in the variables of the dataset (Fig. 3b and Methods). We then examined whether nanocrystal dimensions influence the enhancement by dividing the dataset into smaller (12–14 nm) and larger (17–19 nm) nanocubes. Dramatic differences appear in the change in the PLQY, with the smaller nanocubes improving sharply from 39% to 53% after the first enhancement addition but remaining constant for each further step (Fig. 3c(ii)). By contrast, the PLQY of the larger nanocubes increases gradually, rising from 28% to 36% after the first addition and progressing with each subsequent enhancement step, reaching parity with the smaller nanocubes (53%) after the final step. Similar trends are observed for the primary drivers of PLQY increase, maximum emission and ON percentage, although notably with an onset at different addition steps (Fig. 3c(iii),(iv)). The same qualitative trends are observed when the data are grouped by the surface area subset (Extended Data Fig. 2a). Differences in enhancement dynamics probably reflect variations in the absolute surface area, with larger cubes demanding higher concentrations of repair materials. To further clarify the enhancement mechanism, we isolated the effects of each component (PbBr_2_, OAm and OAc) by introducing them individually to the nanocrystal dispersions. We find that OAm has, by far, the greatest influence (>1,200 nanocubes shown in Fig. 3e; Extended Data Fig. 2c,d shows the data for OAc and PbBr_2_). Adding OAm increased the sample-averaged PLQY from 31.2% to 58%, before decreasing at higher concentrations. This can be explained by the OAm initially shifting the dynamic ligand equilibrium towards the bound state, thereby enhancing surface trap passivation and resulting in higher PLQY values^34^, whereas excessive OAm disrupts the crystal structure^36^. Deconvoluting the single-particle emission metrics (Fig. 3e,f) and the absence of dark cubes in iSCAT images allows us to revisit a long-standing hypothesis: the addition of OAm does not increase the ensemble PLQY primarily due to extending nanocrystal ON times or increasing the fraction of emissive cubes^34,37,38^; instead, it increases the maximum emission of the individual cubes (Supplementary Section 9).

Next, when separating the OAm data into subsets based on the nanocube surface area (Extended Data Fig. 2b), we observe that in contrast to the enhancement solution containing both salt and ligands, the PLQY increase (and decrease) follows the same trajectory across all subsets. Furthermore, the relative PLQY change per ligand addition step remains nearly constant across nanocubes with different surface areas (Supplementary Fig. 17). These observations suggest that ligand coverage is mainly governed by the global ligand equilibrium, resulting in uniform coverage across cubes rather than being determined by individual crystal properties. It follows that the density of unpassivated sites is effectively constant across the population, whereas the absolute number of such sites scales with surface area. As a result, all nanocrystals follow comparable enhancement (and degradation) dynamics, with each ligand addition yielding a similar relative effect on the passivation state. Finally, combining the results of the OAm and enhancement measurements allows us to rationalize the anticorrelation of PLQY and size observed in pristine samples (for example, Figs. 2 and 3c): treatment with PbBr_2_-containing enhancement solution eliminates this size-dependent offset (Fig. 3c), whereas ligand-only treatment does not (Extended Data Fig. 2b and Supplementary Fig. 17). This convergence of PLQY on vacancy repair identifies halide-vacancy-mediated non-radiative recombination as the dominant origin of size dependence. The relationship between PLQY and nanocrystal size—and consequently the effect of halide vacancies on PLQY—is non-trivial, as the interplay of several effects results in intermediate scaling laws and effective power-law exponents between surface and volume effects (Supplementary Section 10). Overall, the combined analysis of nanocrystal dimensions, PL and PLQY indicates that PbBr_2_ in the enhancement solution plays a more critical role for larger cubes, which are more strongly affected by defects arising from halide vacancies.

## Particle size during photoinduced degradation

**Fig. 4:**
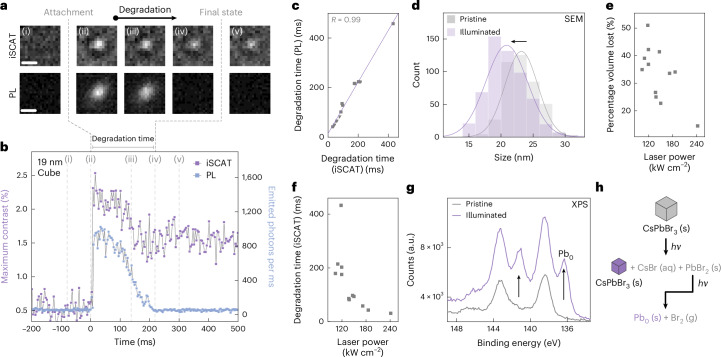
Tracing size reduction and photobleaching of individual sub-20-nm perovskite nanocrystals during photoinduced degradation in a solution environment at millisecond temporal resolution and subnanometre sensitivity. **a**, iSCAT and PL images of a 19-nm perovskite nanocube attaching to a coverglass in hexane during 450-nm illumination (130 kW cm^−2^). iSCAT images have been processed using a temporal median approach. Scale bars, 350 nm (iSCAT); 500 nm (PL). The images clearly show the attachment of a nanocube, followed by a decrease in contrast in both imaging channels, with a complete loss of PL, whereas the iSCAT signal plateaus. **b**, Corresponding degradation traces to the images shown in **a**, confirming that although the emission properties are entirely lost, an iSCAT signal remains after the degradation process. Furthermore, the time of signal loss in the iSCAT and PL channels is similar. The iSCAT contrast in these measurements appears white because of a different focus position and Gouy phase shift^41^. **c**, Scatter plot of degradation time of the individual nanocubes in iSCAT and PL. The strong linear correlation (*R* = 0.99) shows that the signal loss in iSCAT is closely linked to the loss in emission properties, and vice versa. **d**, SEM size distribution of a monolayer nanocube sample with a pristine (*n* = 412; avg = 23.1 nm) and an illuminated (*n* = 569; avg = 20.8 nm) region, showing a size decrease of several nanometres after light exposure in the solution environment, indicating that the main contribution to the iSCAT signal loss is size reduction and not a change in the material refractive index. **e**, Scatter plot of the volume the individual nanocubes lost during degradation given in percentage versus laser power, showing an inverse relationship, with higher laser powers correlating with less volume lost. **f**, Scatter plot of the iSCAT degradation time of the individual nanocubes versus the laser power, showing an inverse relationship. **g**, XPS spectra of the pristine and illuminated region of the monolayer sample used for **d**, clearly showing the emergence of metallic lead during the degradation process. **h**, Proposed degradation mechanism^10,42^. The emitting CsPbBr_3_ nanocube degrades during illumination to a smaller, non-emitting CsPbBr_3_ cube. The side products are CsBr and PbBr_2_, which can further degrade to metallic lead and bromine gas.

Material stability remains one of the greatest challenges for perovskite-based devices^39^, with the underlying degradation dynamics still poorly understood, especially under operating conditions and illumination^40^. Here we use iSCAT to track size reduction and photobleaching in individual nanocrystals, independently of their emission state, under continuous illumination in a solution environment, with millisecond temporal resolution and subnanometre sensitivity. We use the 450-nm laser for both excitation and iSCAT detection, and apply a temporal median background subtraction, where the iSCAT signal remains visible in the processed images throughout particle attachment to the coverglass (Supplementary Section 2b). In a typical experiment, for a 19 nm-sized nanocube (Fig. 4a), the binding appears instantaneously (ii), after which both signals rapidly decrease within hundreds of milliseconds ((iii) and (iv)). In the final state (v), the PL signal vanishes completely, whereas the iSCAT signal assumes a constant, reduced value even under continued illumination (Supplementary Fig. 19 and Supplementary Video 2 show 11 more examples). The kinetic traces reveal near-synchronous onset and the completion of iSCAT degradation and PL bleaching (Fig. 4b), with a strong correlation across 12 cubes (*R* = 0.99; Fig. 4c and Supplementary Fig. 19). The residual iSCAT signal confirms that the nanocrystal has not been entirely destroyed and that a non-luminescent, dark particle persists. This raises questions about the size, composition and origin of the remnant, the reason for halting the degradation and the cause of PL quenching. To address these questions, we first conducted scanning electron microscopy (SEM) measurements and simulations to confirm that changes in the iSCAT signal are primarily driven by the nanocrystal size reduction (Fig. 4e and Supplementary Figs. 21 and 24–26). Unexpectedly, nanocrystals do not degrade completely and exhibit a broad range of volume loss (Fig. 4e, 15%–50%). The percentage of volume loss is largely independent of the initial nanocrystal size (Supplementary Fig. 27), yet is related to the excitation conditions (Fig. 4e), where, counterintuitively, higher laser powers result in less volume lost. To rationalize this, we consider the iSCAT process dynamics. We expected that higher laser powers would accelerate the degradation rate (material loss per millisecond), and shorten the time to reach the same, fixed end-point. However, at higher laser powers, an increased degradation rate (~4×; Supplementary Fig. 20) is outweighed by a larger decrease in degradation time (~8×; Fig. 4f), resulting in less material loss (Fig. 4e). In other words, laser power not only determines the speed of degradation but—through power-dependent quenching—also dictates the end-point of the degradation process. Using X-ray photoelectron spectroscopy (XPS) mapping, we link this behaviour to Pb^0^ formation (Fig. 4g,h and Supplementary Section 12b). Combined with the observation that PL quenching coincides with the halt in size reduction (Fig. 4a), we propose that power-dependent Pb^0^ accumulation suppresses degradation by trapping photoinduced electrons and thereby inhibiting radiative recombination. At high laser powers, rapid Pb^0^ build-up quenches degradation, whereas at lower powers, degradation proceeds for longer, possibly due to incomplete PbBr_2_ decomposition or Pb^0^ diffusion into the solvent (Supplementary Section 15).

## Outlook

We introduce an all-optical technique for characterizing the functionality of individual nanocrystals at a large scale. This non-invasive in situ method enables the simultaneous determination of size and emission of thousands of luminescent nanocrystals within minutes, allowing the high-throughput determination of PLQY at the single-particle level. We observe size-dependent phenomena in the life cycle—that is, from birth to death—of archetypical CsPbBr_3_ perovskite nanocrystals, which were previously masked by bulk averages. In the as-synthesized colloidal sample, we observe a large spread in PLQYs, even among nanocrystals of the same size, with smaller nanocrystals exhibiting higher PLQYs. During post-synthesis solution-phase treatments, we find that larger nanocrystals require additional PbBr_2_ treatment to match the PLQY of their smaller counterparts, indicating that halide vacancies are the leading cause of the size-dependent PLQY differences. In degradation studies, continuous illumination at high laser powers reveals that although the PL signal is rapidly quenched, a substantial fraction of the original nanocrystal remains. We reveal a strong correlation between photobleaching and volume loss, with degraded nanocrystals retaining their cubic shape and forming metallic lead (Pb^0^). Counterintuitively, increasing the laser power quenches the degradation process, which we attribute to the formed Pb^0^ acting as an electron acceptor and absorbing the photoinduced electrons. By isolating single-particle effects within colloidal ensembles, our method provides a pathway towards more efficient and stable perovskite nanomaterials and devices. We anticipate that scaling up single-particle methods will be a cornerstone for designing next-generation nanoparticle-based technologies.

## Methods

### Materials

Caesium carbonate (99%), PbBr_2_ (≥98%), OAc (technical grade, 90%), OAm (technical grade, 70%), 1-octadecene (for synthesis), fluorescein (for fluorescence, >95%) and sodium hydroxide (reagent grade, ≥97%) were purchased from Sigma-Aldrich. *n*-Hexane (for high-performance liquid chromatography, >97%) was purchased from VWR Chemicals. 3-Isocyanotopropyltrimethoxysilane (92%) was purchased from Gelest. FluoSpheres (0.1 μm, 580/605) were purchased from Thermo Fisher. All chemicals were used as received, without further purification.

### Steady-state ultraviolet–visible absorbance and PL

Absorbance and PL spectra were measured on a commercial FluoroMax-4Plus spectrometer equipped with a xenon arc lamp and an F-3031 transmission accessory (HORIBA Scientific). The excitation wavelength for the PL spectra was set to 450 nm and the samples were analysed in quartz cuvettes (Hellma Analytics).

### Bulk PLQY measurements

The absolute PLQY values of diluted colloidal CsPbBr_3_ nanocubes (optical density, ≤0.1) were, if not otherwise stated, determined with a Quanta-φ F-3029 integrating sphere (HORIBA Scientific) by using the direct excitation method, and then corrected for indirect excitation according to ref. ^43^. The excitation wavelength for PLQY measurements was set to 450 nm.

### SEM

SEM images were recorded using a ZEISS SEM Ultra Plus, operating at an acceleration voltage of 3 kV. To enhance the conductivity of the thin-film samples and improve the SEM image contrast, a layer of gold/palladium was sputtered on top using a Leica EM SCD005. The sputtering process was conducted at a current of 15 mA for 25 s, resulting in a 2-nm metal layer deposited on the prepared thin film.

### XPS

The XPS measurements were carried out with a Thermo Fisher Nexsa G2 equipped with a monochromatic (Al Kα) X-ray source. The sample charging was avoided by using a flood gun. High-resolution spectra were performed with a pass energy of 20 eV. The step size was 0.1 eV. The data were evaluated using CasaXPS (v.2.3.26PR1.0). When necessary, charge compensation was performed based on the adventitious carbon assigned at 284.8 eV.

### TEM

TEM imaging was performed on a JOEL JEM-1100 TEM device operated at an acceleration voltage of 80 kV. Specimen preparation was carried out by drop casting 5 µl of diluted colloidal samples onto TEM grids (Electron Microscopy Sciences, Cu with 10/1 nm formvar/carbon). For high-throughput image analysis and automated determination of particles sizes, the ParticleSizer plug-in of the open-source program FijiJ (v.2.16.0) was applied to the images^44^.

### Tip-sonication synthesis of CsPbBr_3_ nanocubes

Cube-shaped CsPbBr_3_ nanocrystals were synthesized in a one-pot approach according to the tip-sonication procedure first introduced in ref. ^26^. In a typical synthesis, 1-octadecene (10 ml) is thoroughly mixed with OAm (0.5 ml) and OAc (0.5 ml), and then caesium carbonate (0.1 mmol, 32.58 mg) and PbBr_2_ (0.3 mmol, 110.1 mg) were added. The reaction mix is immediately subjected to tip sonication (30 W, 10 mins, SONOPULS HD 3100 with a SONOPULS MS73 titanium probe, Bandelin). Crude nanocrystal dispersions were then centrifuged (10,777*g*, 10 min) to remove the remaining unreacted precursor material. The supernatant was discarded and the yellow precipitate, containing CsPbBr_3_ material, was redispersed in *n*-hexane (5 ml) and centrifuged (533*g*, 10 min) for a second time to separate both CsPbBr_3_ bulk material and larger nanocrystals (for the samples measured in Fig. 1c and Supplementary Fig. 15, the precipitate of the first centrifugation step was redispersed in an enhancement solution, and for Fig. 1c, further centrifugation and redispersion in hexane were performed to lower the unbound ligand concentrations and to remove the remnants of the synthesis; the measurement was carried out on the day of synthesis; for Supplementary Fig. 15, the measurement was conducted after 1 year of storage and for the sample used in Supplementary Section 12, the first precipitate was redispersed in a mixture of *n*-hexane (4 ml) and enhancement solution (1 ml), and the measurement was conducted after 2 years of storage; the representative TEM images of the used samples are shown in Supplementary Fig. 1). The precipitate was discarded and the supernatant containing cube-shaped CsPbBr_3_ nanocrystals was kept and stored in ambient conditions. We note that for the measurements shown in Figs. 2 and 3, Extended Data Figs. 1 and 2 and Supplementary Fig. 16, the same stock solution from a single synthesis batch was used. Over time, the solution aged, with the bulk quantum yield increasing from 26% after synthesis to 44% at the conclusion of the measurements. Single-particle measurements started 2 weeks after synthesis and were conducted over a 1-month period, with bulk measurements (Supplementary Fig. 16) performed 7 months later. To minimize accelerated aging effects due to dilution, all the samples were diluted and measured on the same day.

### Coverglass surface functionalization

Coverglasses (Schott D 263 M Glass; #1.5H) were cleaned in an ultrasonic bath with 5-min cycles in Decon90, water, acetone and ethanol. They were subsequently immersed in 3 ml of an 3-isocyanotopropyltrimethoxysilane/toluene solution (1.5 μl of 3-isocyanotopropyltrimethoxysilane per ml of toluene) for 2 h, followed by a rinse with toluene.

### Enhancement and saturated PbBr_2_ salt solution

The PbBr_2_ enhancement solution was prepared by dissolving PbBr_2_ (0.1 mmol, 36.7 mg) in *n*-hexane (10 ml), OAc and OAm (100 µl each) under stirring at 85 °C for up to 3 h until a clear solution is obtained. The enhancement solution was cooled down to room temperature and stored under ambient conditions. The saturated PbBr_2_ solution was prepared by adding an excess of PbBr_2_ salt to hexane and stirring the mixture for 1 h. Atomic emission spectroscopy determined the concentration of PbBr_2_ in hexane to be 4 ppm, after transferring the solute to a water phase for measurement.

### Sample preparation for XPS and SEM measurements

A functionalized coverglass was immersed in hexane (2.5 ml) to which 30 µl of an undiluted nanocrystal solution was added. After an incubation time of 1 h, a marked spot was illuminated by a 450-nm continuous-wave laser (110 kW m^−2^) for 20 min. The solution was removed and the coverglass with the attached and partly illuminated nanocubes was transferred first to XPS and then to SEM measurements, and being stored in a vacuum in between.

### Optical setup measurement procedure and in situ measurements

The custom-made sample holder, with the functionalized coverglass at the bottom, was filled with 1.5 ml of hexane. The holder was then mounted, and 10 μl of the 1:100 diluted nanocrystal mother solution was added (15 μl in the case of Fig. 2 and 25 μl undiluted for Supplementary Fig. 15). Afterwards, the holder was sealed to be gas tight. Image acquisition (iSCAT and PL) typically lasted about 2 min after the addition and continued for ten measurement cycles (approximately 20 min). After each measurement cycle, the PL signals of the attached nanocubes was bleached by high-power illumination at *λ* = 450 nm to set up the new measurement. The speed of the nanocube attachment can be, in principle, controlled through nanocrystal concentration. In the case of the in situ measurements, additional substances, such as the enhancement solution, were introduced into the solution via a pipette, with the sample holder remaining mounted all along. The added enhancement solution, as well as the liquid OAm and OAc ligands, was diluted with hexane, ensuring that 10 μl of the mixture contained the amount of mother solution specified in the figure. The saturated PbBr_2_ solution was used without further dilution.

### Optical setup

The optical setup is shown in Supplementary Fig. 2 and is described in the following. The custom-built iSCAT microscope was detailed in a previous study^17^. In this work, a 638-nm laser (Lasertack) was used a the illumination source. The laser wavelength is below the bandgap of the CsPbBr_3_ samples (Fig. 1b(iii)) and is, thus, not absorbed, leaving the PL emission unaltered. By using a ×60 Olympus objective (×60 is defined relative to a standard tube lens of *f* = 18 cm for Olympus objectives) using an imaging lens with *f* = 100 cm, the setup magnification amounts to 333 ($60\times \frac{100\,{\mathrm{cm}}}{18\,{\mathrm{cm}}}=333$). One pixel on the detector of the Photonfocus camera has a size of 10.6 µm × 10.6 µm. Considering the magnification of 333, one pixel in a measured image (one camera detector pixel) corresponds to 31.8 nm ($\frac{10.6\,\upmu {\mathrm{m}}}{333}=31.8\,{\mathrm{nm}}$) on the sample/coverglass surface. The standard field of view for images taken this work was 256 × 256 pixels or 8.2 µm × 8.2 µm on the coverglass. A second laser at 450 nm (Lasertack) was incorporated for excitation of the perovskite samples, along with a fluorescence detection channel. To achieve this, a dichroic mirror (Thorlabs, DMSP605) was introduced in the beam path after the imaging lens. This mirror reflects the iSCAT contributions at 638 nm and transmits the emitted fluorescence from the perovskite nanocubes at ~515 nm into a scientific complementary metal–oxide–semiconductor camera (Teledyne Photometrics, Prime BSI). A long-pass mirror (Chroma, ET460lp) was placed in front of the camera to block remnants of the 450-nm excitation beam, alongside a bandpass filter (Newport, HPM510-50) to isolate the perovskite fluorescence signal. The standard field of view for fluorescence images was 210 × 210 pixel^2^ after 2 × 2 camera-internal software binning, corresponding to 8.2 µm × 8.2 µm on the coverglass (pixel size, 39 nm). The iSCAT acquisition software with live processing was custom-made in LabVIEW NXG, whereas the PL images were acquired using Micro-Manager^45^.

### Image acquisition and analysis

Unless stated otherwise, iSCAT images were acquired at a size of 256 × 256 pixel^2^, with an exposure time of 0.7 ms and at 122 frames per second (fps). In the software, a 7-ms offset was applied between each image acquisition to better align the number of iSCAT and PL images. The iSCAT images presented in Fig. 4 and Supplementary Figs. 19 and 27 were acquired at an exposure time of 0.35 ms and at a speed of 1,267 fps (no software offset). PL images were acquired at a size of 210 × 210 pixel^2^ with an exposure time of 75 ms and a frame rate of 12 fps (100 ms and 10 fps for Supplementary Fig. 15; 1 ms and 303 fps for Fig. 4). The samples were excited at a laser power density (at 450 nm) of 7.5 W cm^−2^ (Fig. 2), 8.0 W cm^−2^ (Fig. 3a), 6.9 W cm^−2^ (Fig. 3b), 8.4 W cm^−2^ (Extended Data Fig. 2), 4.5 W cm^−2^ (Supplementary Fig. 15) and 100–240 kW cm^−2^ (Fig. 4 and Supplementary Figs. 19 and 27). Unless otherwise stated, the iSCAT images were corrected for fluctuations in the laser light intensity and the background was subtracted by the DRA approach (batches of 200 images were averaged for DRA analysis). The background-subtracted images were subsequently spatially binned (2 × 2) and subjected to a column-projection fixed-pattern noise correction. DRA subtraction and the subsequent particle localization were performed using modules of the open-source software PiSCAT^29,30^. Particles were localized based on a difference of Gaussian algorithm, with outliers filtered to produce a dataset containing particle contrast, spatial positions and temporal information. The background-corrected iSCAT images, along with the fluorescence images, were processed using our custom-made correlation script, which automatically correlated and analysed particles across both channels. To facilitate software processing, we periodically calibrated the acquisition setup by aligning the iSCAT and PL channels using the mass centre of the fluorescent beads drop cast onto a glass coverslip, visible in both channels. This initial alignment ensured that iSCAT and fluorescence images shared the same field of view. Minor shifts were subsequently corrected automatically by the correlation software. Detailed information about the script is provided in Supplementary Section 5. For the iSCAT images shown in Fig. 4 and Supplementary Figs. 19 and 27, temporal median background subtraction was performed using the open-source program FijiJ. The median was calculated from the 100 frames preceding the corresponding nanocube event, followed by the application of a fast Fourier transform bandpass filter. The images were then spatially binned (2 × 2), and five successive frames were temporally averaged. For the iSCAT and PL traces, the pixel values around the particle centres were used (10 × 10 pixel^2^).

### PLQY calculation

The calculation of single-particle PLQYs requires determining the amount of absorbed and emitted photons per particle. First, the excitation and detection channel must be calibrated to obtain quantitative information about the absorbed and emitted photons, accounting for transmission losses in the optical beam path, objective collection efficiency and quantum efficiency of the camera (Supplementary Section 4 provides the exact methodology). Following this, the number of emitted photons can be calculated directly from converted PL images. Grey-scale values in the PL images obtained with the calibrated setup can be converted to the number of photons emitted by subtracting a baseline corresponding to the dark counts (ref. ^46^ provides more details):

$${{I}}_{\mathrm{phot}}^{\mathrm{PL}}\left({x},\,{y}\right)={{I}}_{\mathrm{raw}}^{\mathrm{PL}}\left({x},\,{y}\right)-{{I}}_{\mathrm{dark}}.$$

Through single-particle tracking (Supplementary Section 5), the average number of photons emitted in each frame by every particle is extracted by summing up over the area of their PSF:

$${N}_{{\rm{em}}}={\sum }_{{\rm{PSF}}}{I}_{{\rm{phot}}}^{{\rm{PL}}}\left({x}_{0},\,{y}_{0}\right),$$

where $\left({x}_{0},\,{y}_{0}\right)$ indicates the particle position.

For a fair comparison between the iSCAT and PL data, it is essential to account for differences in image normalization. Although iSCAT images are normalized, making the detected contrast and, therefore, volume independent of the incident laser power, raw PL images are not, as the emission is proportional to the number of absorbed photons. Due to the Gaussian profile of the illumination beam, particles at the edges of the field of view exhibit lower PL emission than those at the centre.

To compare the emission of particles located at different positions in the field of view, for example, in the emission versus size plots shown in Fig. 2, we need to consider the non-homogeneous (roughly Gaussian) illumination. To do this, we record an image of the impinging beam and normalize it to its maximum, so that each pixel has a value between 1 and 0. The PL images are then divided by this image and the analysis of the single-particle emission is carried out on the normalized frames. In this way, each particle effectively absorbs the same number of photons as if they were at the centre of the focused laser spot.

Having calculated the number of emitted photons, we need to evaluate the number of absorbed photons per particle, which can be extracted through the particle absorption cross-section. For this estimation, we need the particle volume ${V}_{{\rm{c}}}$, here determined via iSCAT, and the particle dielectric function. The absorption cross-section ${\sigma }_{{\rm{abs}}}$ can be calculated from the imaginary part of the polarizability α as

$${\sigma }_{{\rm{abs}}}=k\times {\rm{Im}}(\alpha ),$$

where $k=2{\rm{\pi }}{n}_{\mathrm{glass}}/\lambda$ is the wavevector. We use ${n}_{\mathrm{glass}}$ because the light is incident from below through the immersion oil and coverglass, which are refractive index matched. In the case of a deeply subwavelength particle in a homogeneous medium, the polarizability is approximately independent of the particle geometry^28^, and can be estimated by that of a sphere:

$$\alpha =4\times {\epsilon }_{{\rm{medium}}}\times \,\uppi \times {r}^{3}\times \frac{\left(\epsilon -\,{\epsilon }_{{\rm{medium}}}\right)}{(\epsilon -2\times {\epsilon }_{{\rm{medium}}})},$$

where r is the sphere radius, ϵ is the dielectric function of the particle (here CsPbBr_3_ (ref. ^47^)) and ${\epsilon }_{{\rm{medium}}}$ is that of the surrounding environment (here hexane^48^). In our calculations, we use an effective radius ${r}_{{\rm{eff}}}$ that results in the same volume as the cubic nanocrystals: ${r}_{{\rm{eff}}}={(3{V}_{{\rm{c}}}/4\pi )}^{1/3}$. In our calculations, we estimate the absorption cross-section of each nanocrystal from its size using a quasistatic polarizability model that assumes a uniform complex dielectric function. Although this approach provides a consistent framework for extracting absorbed photon flux and, ultimately, PLQY, it implicitly assumes negligible variation in the dielectric function (*ε*) across the particle ensemble. For the CsPbBr_3_ perovskite nanocrystals studied here, this assumption is reasonable: the materials are synthesized as single-phase nanocubes without graded interfaces or core–shell structures. Although defects such as halide vacancies or undercoordinated ions may be present, they are unlikely to induce substantial changes in *ε*. Furthermore, the nanocrystals are above the quantum confinement regime, meaning that their dielectric function is not size dependent and no corresponding correction factor is required. They also exhibit negligible anisotropy. Consequently, PLQY variation stemming from dielectric heterogeneity is expected to be minimal in our case. However, in other material systems, fluctuations in the dielectric function may need to be explicitly accounted for when applying this methodology, for example, by introducing a size correction term inside the equation for the dielectric function. Supplementary Section 3d and ref. ^49^ provide a comprehensive discussion of this issue in the context of quantum efficiency measurements. With the particle absorption cross-section, the energy absorbed per frame can be calculated as

$${E}_{{\rm{abs}}}={I}_{{\rm{irr}}}\left({x}_{0},{y}_{0}\right)\times {\sigma }_{{\rm{abs}}}\times \Delta t\,.$$

Here ${I}_{{\rm{irr}}}\left({x}_{0},{y}_{0}\right)$ is the irradiance in ${\rm{W}}\,{{\rm{m}}}^{-2}$ at the particle position and Δt is the exposure time of the camera during the recording of PL images. The image representing the laser irradiance can be obtained in two steps: first, we normalize the image of the laser profile so that the sum over all pixels is equal to the total laser power impinging at the sample (measured separately):

$${I}_{{\rm{power}}}^{{\rm{laser}}}=\frac{{I}^{{\rm{laser}}}}{\sum {I}^{{\rm{laser}}}}\times {P}_{{\rm{laser}}}\,.$$

Then, we divide ${I}_{{\rm{power}}}^{{\rm{laser}}}$ by the area on the sample each pixel in the image corresponds to, which is determined by the ratio of the physical area of the camera pixel ${A}_{{\rm{pix}}}$ to the magnification M of the microscope (here M=333). ${I}_{{\rm{irr}}}={I}_{{\rm{power}}}^{{\rm{laser}}}\times M/{A}_{{\rm{pix}}}$ is then giving an image in ${\rm{W}}\,{{\rm{m}}}^{-2}$.

However, as we described above, given the normalization we adopted for the PL images, the effective irradiance in our processed images is constant over the field of view (to be able to compare the iSCAT and PL data). To obtain this value of irradiance and to obtain the PLQY values unaffected by our normalization, we can simply apply the same normalization procedure used for the PL images to the ${I}_{{\rm{irr}}}$ image:

$${I}_{{\rm{irr}}}^{{\rm{const}}}=\frac{{I}_{{\rm{irr}}}}{{I}_{{\rm{norm}}}^{{\rm{laser}}}}=\frac{\max ({I}^{{\rm{laser}}})}{\sum {I}^{{\rm{laser}}}}\times {P}_{{\rm{laser}}}\times \frac{M}{{A}_{{\rm{pix}}}}.$$

${I}_{{\rm{irr}}}^{{\rm{const}}}$ is the effective flat laser irradiance over the field of view. The energy absorbed by each particle in a frame then solely depends on the volume and not on the position:

$${E}_{{\rm{abs}}}={I}_{{\rm{irr}}}^{{\rm{const}}}\times {\sigma }_{{\rm{abs}}}\times \Delta t\,.$$

Finally, the number of absorbed photons is retrieved by dividing the absorbed energy by the photon energy at the illuminating frequency ν: ${N}_{{\rm{abs}}}={E}_{{\rm{abs}}}/h\nu$.

The PLQY of each particle is obtained by dividing the number of emitted photons by the number of absorbed ones, considering a factor η that corrects for the losses in the optical beam path determined when calibrating the setup, the approximation made in the absorption cross-section calculation by considering a homogeneous environment and variations relative to a reference bulk measurement (Supplementary Sections 4 and 7):

$${\rm{PLQY}}=\eta \frac{{N}_{{\rm{em}}}}{{N}_{{\rm{abs}}}}.$$

For this calculation, the number of emitted photons is taken as the average collected on many frames (N=200) to negate the effect of PL blinking.

### CPA

CPA is a statistical method used to detect points in a time series in which the underlying probability distribution changes. These change points indicate shifts in the data behaviour, such as changes in mean, variance or other statistical properties. In our case, we use CPA to estimate at which point in a blinking trace the transition between two states with different emission properties is found. It leads to a ‘discretization’ of the PL traces, making it easier to extract relevant quantities. Briefly, it uses Bayesian theory to estimate a ‘Bayes factor’ giving the likeliness that in a time series originating from a Poisson distribution, a jump occurred^32,50^. If the Bayes factor exceeds a certain threshold (in this study, 20% of the maximum emission), the trace is divided and the ‘Bayes factor’ calculated again, until there is no sub-trace in which the calculated factor exceeds the threshold value. Clustering into states of the PL trace is obtained by taking the average value between successive change points.

### PCA

PCA reduces the dimensionality of datasets (in our case comprising size, PLQY, maximum emission, average emission, ON percentage, blinking amplitude and switching rate) into uncorrelated principal components (PCs) that can be plotted in a single, two-dimensional graph. Distinct clustering of data points before and after enhancement indicates notable variances in the underlying parameter distributions (for example, in Fig. 3b, due to defect repair). The enhanced sample shows a shift primarily along principal component 1 (PC1, *x* axis) and, to a lesser extent, along principal component 2 (PC2, *y* axis), as evidenced by the centres of mass of the two distributions (Fig. 3b, crosses). Each of the ingoing parameters is represented by an arrow, known as a loading vector, indicating the direction and strength of the original variable’s influence on the PCs. The loading vectors help to quickly identify which features are most strongly associated with the overall enhancement due to defect repair. In Fig. 3b, these are, for example, average emission, maximum emission and blinking amplitude for PC1, switching rate for PC2, and PLQY and ON percentage with contributions to both PCs. Additionally, the loading vectors illustrate correlations between different parameters. For example, the switching rate is inversely correlated with size (opposite direction of arrows) but decoupled from other variables such as blinking amplitude (perpendicular arrows). This suggests that these variables reflect distinct aspects of the material’s properties and, therefore, different photophysical origins.

### Lumerical simulations

Optical simulations are performed with a commercial software (Lumerical 2023 R2.3) used to solve Maxwell’s equations in the time domain. A total-field–scattered-field source with perfect-matched-layer absorbing boundary conditions is used to isolate the contribution from particle scattering. Symmetry conditions are used in the x−y directions to reduce the simulations size to 1/4th the total volume (set to $20\,\upmu {\mathrm{m}}\times 20\,\upmu {\mathrm{m}}\times 0.6\,\upmu {\mathrm{m}}$). Light is incident from below the glass substrate and a cubic particle is located 2 nm above the glass–hexane (n=1.375) interface to account for the length of the functionalization group and ligands present on the surface. We use a dense mesh around the cubic particle that is double its size with a mesh spacing 1/10th the nanocube side length. To reproduce the normalization procedure used in iSCAT, we perform a simulation with the particle and one without, keeping everything else fixed. Optical imaging is simulated by performing the far-field projection of the fields collected by a monitor positioned at the glass–hexane interface. The far-field projection is done through the ‘microscopy_imaging’ script available from the Lumerical knowledge base, which considers the numerical aperture and the defocusing of the objective. To obtain the simulated iSCAT images, we perform the ratio between the images collected with and without the particle. The defocusing parameter is adjusted to maximize the particle contrast (negative or positive), as during experimental alignment, the relative sample–objective distance is fixed in the same way. This procedure corresponds to finding the position in which the reflected light by the coverglass and the one scattered by the particle constructively or destructively interfere. The amount of defocusing needed to achieve this condition is determined by the scattering phase delay and the Gouy phase accumulated between the scattered and reflected components.

## Online content

Any methods, additional references, Nature Portfolio reporting summaries, source data, extended data, supplementary information, acknowledgements, peer review information; details of author contributions and competing interests; and statements of data and code availability are available at 10.1038/s41563-026-02607-5.

## Supplementary information

Supplementary Information Supplementary Figs. 1–27, Sections 1–15 and Tables 1 and 2.

Supplementary Video 1 Correlating iSCAT and PL signals of single perovskite nanocrystals. Attaching nanocrystals result in black and white signals in the iSCAT and PL channels, respectively. In iSCAT, the signal of the attached cubes is continuously subtracted as background, using a DRA background subtraction method. This approach substantially enhances the measurement signal-to-noise ratio through the averaging of multiple frames. As a result of this analysis, the iSCAT signal of a particle appears in the corrected images only for a short duration on its attachment to the coverglass. Conversely, in PL microscopy, the emission signals from attached cubes accumulate over time. Correlating the two channels enables the calculation of the PLQY of the individual particles. iSCAT images were acquired at a speed of 8.2 ms per frame (122 fps) and background-subtracted (DRA, 200 images per batch). The contrast is adjusted from –0.0035 to 0.0049. PL images were acquired at a speed of 75 ms per frame (12 fps). Scale bar, 2 µm (all images).

Supplementary Video 2 Simultaneous iSCAT and PL video of a single perovskite nanocrystal attaching and degrading under high-power illumination. After the attachment of the nanocrystal, a signal appears in the PL and iSCAT channels. Over time, the PL signal degrades completely, whereas in iSCAT, the signal is reduced but remains at a lower intensity. The iSCAT images were acquired at a speed of 0.78 ms per frame (1,282 fps), background subtracted (temporal median), 2 × 2 binned and temporally averaged (five frames). The contrast is adjusted within 0.99–1.02. Scale bar, 350 nm. PL images were acquired at a speed of 3.3 ms per frame (303 fps) and 2 × 2 binned (camera internal). Scale bar, 500 nm. The video is slowed down such that 1 s in this video equals 156 ms in real time (total real time of video, 1.56 s).

## Data Availability

All data necessary to support the conclusions are available in the Article or its Supplementary Information and are publicly available via Zenodo at 10.5281/zenodo.19552115 (ref. ^51^).

## Code availability

The code used in this work is available from the corresponding authors upon reasonable request.
