# Supplementary material for: High-throughput in situ sizing and quantum yield determination of individual perovskite nanocrystals
Source: Nat Mater. 2026 May 21;25(8):1344–51. doi: 10.1038/s41563-026-02607-5 (PMC13421312; doi:10.1038/s41563-026-02607-5)
Supplement: Supplementary file 1 — Supplementary Figs. 1–27, Sections 1–15 and Tables 1 and 2. [file 41563_2026_2607_MOESM1_ESM.pdf]

nature materials

Supplementary information https://doi.org/10.1038/s41563-026-02607-5

High-throughput in situ sizing and quantum yield determination of individual perovskite nanocrystals

In the format provided by the authors and unedited

Contents

Supplementary video guide 4
Section 1. TEM images of samples used 5
Section 2. iSCAT methodology 6
a) iSCAT signal origin and contributions 6
b) Background correction and image processing 7
Section 3. Errors, uncertainties, variations associated with the measurements of particle volume and quantum efficiency 10
a) General discussion on errors and uncertainties 10
b) Agreement of iSCAT and TEM distributions and Calibration Fit 12
c) Error estimation 14
d) Dielectric function and possible variations 15
Section 4. Calibrating setup for quantitative emission, excitation and PLQY measurements 17
a) Setup calibration 17
b) Laser power excitation in the linear regime 18
Section 5. Python script analysis and workflow 19
Section 6. PLQY reference bulk 24
Section 7. Bulk treatments with enhancement solution and oleylamine 27
Section 8. Relative PLQY increase per OAm addition step 29
Section 9. Mechanism of OAm enhancement 30
Section 10. Anticorrelation of PLQY and size in pristine samples 31
Section 11. Degradation of single cubes traced simultaneously by iSCAT and PL 35
Section 12. SEM and XPS measurements on sample with illuminated and pristine cube region 37
a) SEM 37
b) XPS 39
Section 13. Lumerical Simulations – 450 nm and degradation study 42
a) iSCAT contrast-to-volume formula at 450 nm illumination 42
b) Simulations on impact of a loss of absorption properties or a transformation into $PbCO_3$ on the iSCAT contrast 43
Section 14. Volume dependence of degradation 44
Section 15. Minimal degradation model 45
References 47

Supplementary video guide

Supplementary Video 1. Correlating iSCAT and PL signals of single perovskite nanocrystals.

Attaching nanocrystals result in black and white signals in the iSCAT and PL channels, respectively. In iSCAT, the signal of the attached cubes is continuously subtracted as background, using a differential rolling average background (DRA) subtraction method. This approach significantly enhances the measurement signal-to-noise ratio through the averaging of multiple frames. As a result of this analysis, the iSCAT signal of a particle appears in the corrected images only for a short duration upon its attachment to the coverglass. Conversely, in PL microscopy, the emission signals from attached cubes accumulate over time. Correlating the two channels enables the calculation of the PLQY of the individual particles. iSCAT images were acquired at a speed of 8.2 ms per frame (122 fps) and background-subtracted (DRA, 200 image batch). The contrast is adjusted to -0.0035 - 0.0049. PL images were acquired at a speed of 75 ms per frame (12 fps). Scale bar, 2 µm (applies to all images).

Supplementary Video 2. Simultaneous iSCAT and PL video of a single perovskite nanocrystal attaching and degrading under high power illumination.

After the attachment of the nanocrystal, a signal appears in the PL and iSCAT channel. Over time the PL signal degrades completely, while in iSCAT the signal is reduced but remains at a lower intensity. iSCAT images were acquired at a speed of 0.78 ms per frame (1282 fps), background-subtracted (temporal median), 2×2 binned and temporally averaged (5 frames). The contrast is adjusted to 0.99 - 1.02. Scale bar, 350 nm. PL images were acquired at a speed of 3.3 ms per frame (303 fps) and 2×2 binned (camera internal). Scale bar, 500 nm. The video is slowed down, so that 1 s in this video equals 156 ms in real time (total real time of video: 1.56 s).

Section 1. TEM images of samples used

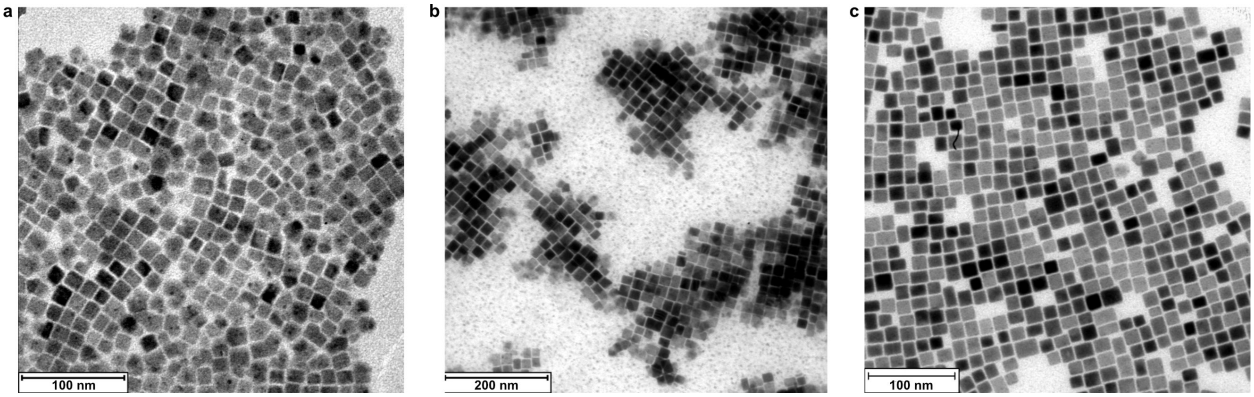

Supplementary Figure 1. Representative TEM images of the samples measured (it needs to be noted that perovskite nanocrystals were observed to self-heal over time but overall maintained the cubic shape and size). For more details, see also methods. **a** TEM image of the sample used in measurements presented in Fig. 2, 3 and Extended Fig. 1,2 and Supplementary Figure 16. Image was taken on first day of the single particle measurement row which lasted for 1 month. **b** TEM image of the sample used in measurements presented in Fig. 1c and Supplementary Figure 15. Here, the cubes were redispersed in enhancement solution during the preparation (see methods). Image is taken on the day of synthesis which is also the measurement day of the data presented in Fig 1c (before the measurement, the sample was again centrifuged and redispersed in hexane). In case of Supplementary Figure 15, the sample was measured as is, however after storage of 1 year. **c** TEM image of the sample used in measurements presented in Supplementary Information Section 12. Here, the cubes were redispersed in a mixture of n-hexane (4 mL) and enhancement solution (1 mL) during the preparation (see methods). They were measured after 2 years of storage. The image was taken on the measurement day.

Section 2. iSCAT methodology

a) iSCAT signal origin and contributions

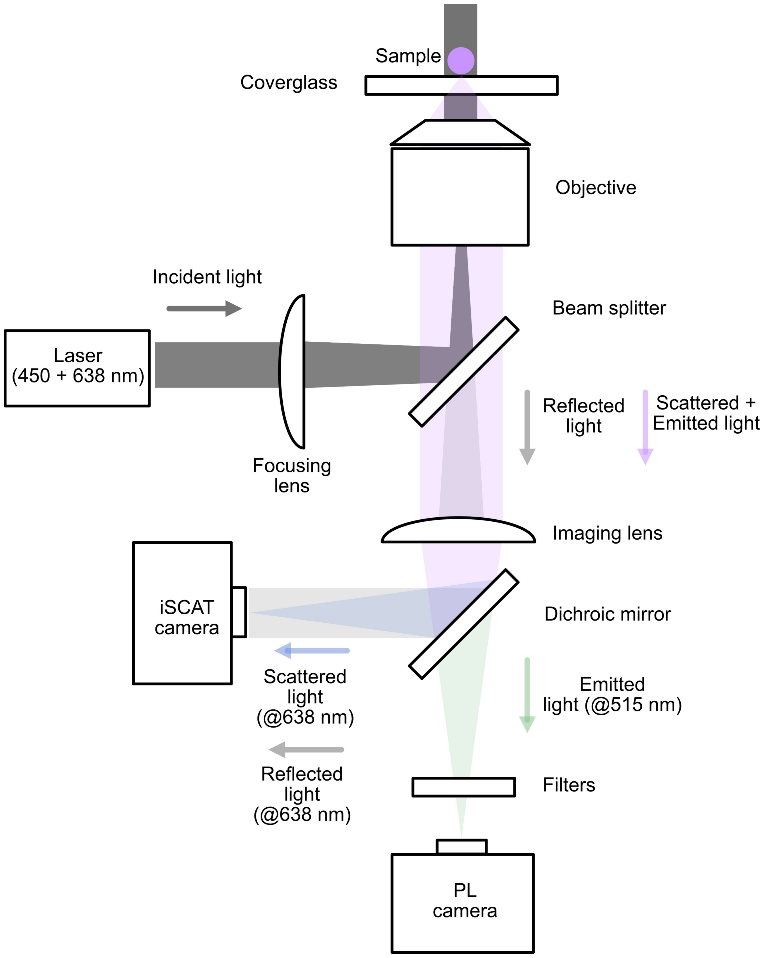

Supplementary Figure 2. Schematic overview of the beam path of the custom-built iSCAT and PL microscope used in this study.

Supplementary Figure 2 presents the optical layout of the optical microscope employed in the experiments. For the interferometric scattering channel, the detected light ($I_{detected}$) at the iSCAT camera is the result of the superposition of the reflected light field ($E_{reflected}$) and the scattered light field ($E_{scattered}$), as expressed in Equations 1.1 and 1.2:

$$I_{detected} = \left|E_{reflected} + E_{scattered}\right|^2 = \quad \text{(Equation 1.1)}$$

$$= \left|E_{reflected}\right|^2 + |E_{scattered}|^2 + 2\left|E_{reflected}\right||E_{scattered}|\cos(\Delta\phi) \quad \text{(Equation 1.2)}$$

Reflected light is generated by the refractive index mismatch at the interface between the coverglass and the sample medium. This component does not carry information about the particles scattering, is constant, and can be subtracted to isolate the signal from the sample (see for more details Section 1 b). The scattering signal encodes information about the sample as it arises from

changes in the local refractive index caused by events like the attachment of a particle. The scattered light field is proportional to the complex polarizability α of the scatterer:[1]

$$E_{scattered} \propto \alpha E_{incident} \qquad \text{(Equation 2)}$$

In the quasistatic approximation for sub-wavelength particles, α is given by:

$$\alpha = 3\epsilon_m V \left(\frac{\epsilon_s - \epsilon_m}{\epsilon_s + 2\epsilon_m}\right) \qquad \text{(Equation 3)}$$

where V is the particle volume, and ϵ_s and ϵ_m represent the permittivities of the scatterer and the medium, respectively. The polarizability α primarily depends on the particle's volume V,[1] which scales with the cube of its diameter (d^3).

In the camera signal, there are two contributions from light scattering: a pure scattering term ($|E_{scattered}|^2$, used for dark-field microscopy) and an interferometric scattering term ($2|E_{reflected}||E_{scattered}|\cos(\Delta\phi)$). For small scatterers (d < 50 nm), the pure scattering term is negligible due to its sixth-power scaling with scatterer diameter (halving the diameter reduces the signal by a factor of 64), unlike the third-power scaling of the interferometric term. As a result, small scatterers primarily contribute through the interference term, where the reflected light acts as a reference. This interferometric signal manifests as small variations on the large reflective background and is revealed only after image analysis. This signal in iSCAT imaging is tied to the scattered light field and, consequently, to the polarizability α, which depends on the particle's size and refractive index. This dependency makes it possible to extract the particle's volume and size, while the third-power scaling of the signal with diameter allows for the detection of extremely weak scatterers.

When scatterers grow larger, the pure scattering contribution becomes significant and eventually dominates the signal due to its stronger scaling with diameter. Therefore, a linear relationship between contrast and volume can only be established for small particles for which the pure scattering term is negligible. This shift can be observed experimentally, for instance, during nucleation seed growth,[2] where the signal changes from negative/black to positive/white due to the opposing contributions in Equation 1.2. Note that focus position can also invert the interferometric signal for small scatterers to a positive/white contrast because of the Gouy phase shift.[3]

b) Background correction and image processing

Static background features in raw iSCAT images need to be eliminated by background correction. These features arise from sources such as reflections at the coverglass interface, optical component impurities, and spurious back-reflections.[4] Two common background subtraction methods, differential rolling average (DRA) and temporal median subtraction, were employed in this study (see Supplementary Figure 3).

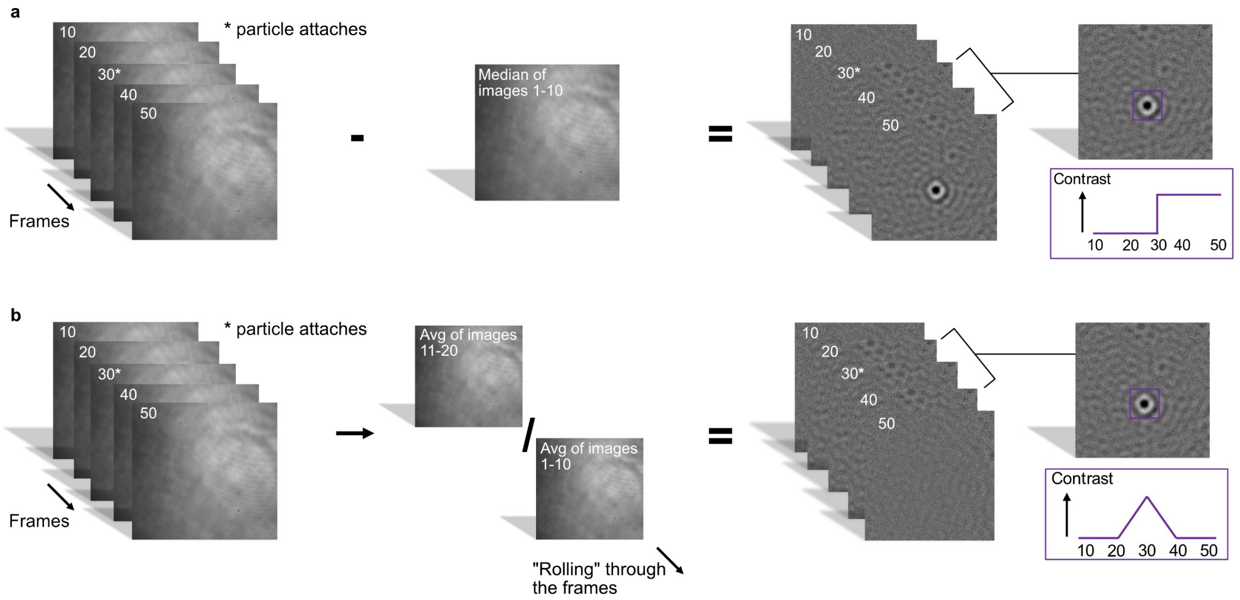

Supplementary Figure 3. Background subtraction in iSCAT image processing. **a** Principle of the temporal median approach. Dividing the raw images by the temporal median image of the first 10 frames eliminates static background features that mask the dynamic reaction processes (here, the attachment of a particle at frame 30). **b** Principle of the differential rolling average approach. Subsets of images are averaged to create the reference image and the raw image, that are subsequently divided pixel-wise. This process is applied in a rolling manner throughout the image stack. In case of an attaching particle, this results in frames with increasing, peaking, and decreasing particle contrast, with the peak representing the particle's true signal.

Temporal Median Subtraction:

This method involves calculating the median pixel intensity for each pixel across consecutive raw images (100 in this study) acquired prior to the event of interest (e.g., particle attachment). The resulting median image represents static background features. Dividing each raw image in the stack by this median image eliminates static components, leaving only accumulated changes over the acquisition period. For instance, when a particle attaches, its contrast becomes apparent in the corresponding frame and remains visible as long as the particle stays (Supplementary Figure 3b).

DRA:

The DRA approach involves creating reference and raw images by averaging subsets of frames within the image stack. These subsets are rolled forward through the stack, and the raw images are divided by the reference images, isolating dynamic changes. Averaging subsets of images (200 frames in this study) significantly reduces noise, as discussed later. In the context of particle attachment, this background correction method may give the illusion that the particle's contrast rises and then falls, whereas the particle is, in fact, stationary after attachment.

Additional Image Processing:

An intrinsic noise source in iSCAT measurements is shot noise, caused by statistical variations in the number of photoelectrons (N) detected by the camera.[5] Shot noise scales with $\sqrt{N}$ and can be reduced by increasing the illumination intensity and thus the number of detected photoelectrons. However, practical constraints, such as the camera's full well depth, limit this approach. Two additional techniques were employed after background subtraction in this study to minimize this noise: pixel binning and temporal averaging.

In pixel binning, neighboring pixels are averaged electronically to increase the number of detected photoelectrons per pixel. For all images in this study, unless noted otherwise, 2x2 pixel binning was applied, where four adjacent pixels were averaged into a single pixel. This process reduced the image resolution from 256x256 to 128x128 pixels but increased the effective number of photoelectrons fourfold, reducing shot noise by half ($\sqrt{4N}$).

Temporal averaging averages pixel values over consecutive images instead of within a single image. Where noted, five consecutive frames were averaged into one, with each pixel representing the average intensity at the same location across these frames. This method reduced shot noise by the square root of five ($\sqrt{5N}$) at the cost of temporal resolution.

Section 3. Errors, uncertainties, variations associated with the measurements of particle volume and quantum efficiency

a) General discussion on errors and uncertainties

Since the extraction of the quantum yield involves several steps, it is challenging to provide a precise quantitative assessment of the overall uncertainty for each particle. Nevertheless, we offer quantitative estimates where possible and indicate the main sources of error. The total uncertainty on the quantum yield can thus be decomposed into contributions from the following steps:

- Correlation of TEM size with iSCAT contrast ("Calibration")
- Evaluation of the number of absorbed photons
- Evaluation of the number emitted photons

Correlation of TEM size with iSCAT contrast ("Calibration") - Systematic errors in the contrast-to-volume calibration will propagate directly into the quantum yield determination. *TEM:* A potential error source is that TEM yields only the in-plane projection of each nanocube; following common practice,[6] the out-of-plane dimension is therefore approximated as the mean of the two in-plane dimensions. To minimize human error in the TEM-based volume estimation, particle sizes were determined using a semi-automated routine (ParticleSizer in the Fiji image analysis software). Deviations from ideal cubic shape could, in principle, introduce systematic errors in the estimated volume. In our case, several aspects mitigate this concern. First, the synthesis route employed (tip-sonication method) produces predominantly cubic $CsPbBr_3$ nanocrystals, consistent with both our TEM analysis and literature reports. We have quantitatively examined this potential source of uncertainty using our full TEM dataset. Across 1033 nanocrystals, the projected in-plane aspect ratio (long/short axis) is 1.1568 ± 0.1360 (mean ± s.d.). The distributions of the two lateral dimensions are unimodal, with no evidence of bimodality or a distinct anisotropic subpopulation, as would be expected, for example, for platelet-like particles that typically produce bimodal size distributions because the short thickness dimension enters the measured size distribution through upright oriented platelets.[7] This confirms predominantly quasi-cubic morphology and rules out a significant population of strongly elongated rod- or platelet-like structures. Second, the TEM-based size distribution used for calibration is derived from more than 1000 particles and analyzed statistically. The conversion between iSCAT contrast and volume is not based on individual particle matching but on percentile correlation of entire distributions (Fig. 1c). After applying this conversion, the resulting iSCAT size distribution shows excellent agreement with the independently measured TEM size distribution ($R = 0.99$). This empirical validation demonstrates that any residual deviations from ideal cubic geometry introduce at most minor statistical broadening and do not materially affect the contrast-to-volume conversion. *iSCAT:* Uncertainties may arise from focus variations during iSCAT measurements, from errors in fitting the point spread function resulting in an incorrect evaluation of the iSCAT contrast, and from scattering fluctuations due to locally heterogenous coverglass roughness. In particular, strong baselines in the background signal can lead to inaccuracies in the determination of the particle contrast (see https://piscat.readthedocs.io/Tutorial4/Tutorial4.html) in the DRA traces, which propagate in the calibration curve. We therefore make sure the impinging beam is roughly uniform at the image

plane without significant distortions. These measurement-intrinsic and instrument-related errors are expected to amount to around 5-10% and are discussed in greater detail in Section 3b, c. *Calibration Fit:* A highly linear correlation between iSCAT contrast and particle volume can be obtained experimentally with large datasets, yielding a calibration uncertainty below 2% in our case. This, however, does not imply that single-particle volume estimates are equally precise; it simply ensures that the conversion from iSCAT contrast to volume does not add further uncertainty. This is discussed in greater detail in Section 3b, c.

Evaluation of the number of absorbed photons - One potential source of systematic error is the use of the absorption cross-section formula for a homogeneous environment, rather than accounting for the presence of the substrate. However, given that the refractive index of the substrate (glass, $n \approx 1.45$) and environment (hexane, $n \approx 1.38$) is rather close, this effect is expected to be small. The modification of the scattering cross section of a subwavelength particle at a distance z from a substrate can be calculated analytically,[8] which can give us a quantitative estimation of the error. The effective polarizability in the case of an in-plane dipole (normal-incidence excitation) is:

$$\alpha_{eff} = \frac{\alpha(1-\beta)}{1 - \frac{\alpha\beta}{32\pi(r+z)^2}}$$

Where $\beta = (\epsilon_{glass} - \epsilon_{hexane})/(\epsilon_{glass} + \epsilon_{hexane}) \approx 0.05$. As β is small, the second term in the denominator becomes negligible, and the correction to the polarizability is basically $\alpha(1-\beta)$. This corresponds to an approximate 5% overestimation of the polarizability. Notably, this correction factor is essentially independent of particle size and introduces only a systematic shift in the calculated number of absorbed photons. Such a systematic shift is already effectively accounted for in the correction factor derived from the bulk PLQY measurements (see Section 4a).

Another possible contribution of error comes from the deeply subwavelength approximation used for cubic particles; this is expected to be small, since typically $L < \lambda/20$.
Further, laser power fluctuations can influence the local intensity and thus the calculated number of absorbed photons. However, according to the laser manufacturer, power variations below 1 % are expected over timescales of up to 8 h - much longer than the duration of individual measurements in our experiments.
Dielectric heterogeneity, whether systematic or particle-to-particle, may need to be considered depending on the material (see detailed discussion in Section 3d).
Finally, the number of absorbed photons is calculated as $N = E_{\text{abs}}/(h\nu)$; therefore, any error in the laser wavelength directly affects N. According to the manufacturer, the central emission wavelength is stable within ±5 nm, with a typical spectral bandwidth of less than 2 nm. Given our excitation wavelength of 450 nm, this corresponds to a possible systematic error below 1% due to central wavelength shifts and a statistical fluctuation of approximately 0.5% associated with the laser bandwidth.

Evaluation of the number of emitted photons – During particle selection in the analysis software, we minimized potential systematic errors by correcting for offsets between the iSCAT and PL images, excluding overlapping particles, and removing particles that exited the field of view prematurely (see Section 5). These steps ensured that only isolated perovskite nanocubes with a sufficient number of frames were analyzed, thereby minimizing the influence of blinking on the averaged emission and enabling reliable evaluation of the emission time traces.
Additional uncertainties may arise from the correction factor used to account for the optical system's collection efficiency, including possible errors in the calibration factor that compensates for losses along the photoluminescence detection path and the objective's collection angle. These contributions introduce a systematic shift, which is already effectively accounted for in the correction factor derived from bulk PLQY measurements (see Section 4a).
A further source of uncertainty stems from using a single representative value for the camera quantum efficiency (QE) across the nanocubes' PL emission bandwidth. According to the manufacturer, the QE varies by approximately ±2% within this range, introducing a corresponding fluctuation in the calculated number of emitted photons, which are assumed to be detected at the average QE.

b) Agreement of iSCAT and TEM distributions and Calibration Fit

Distribution widths:

iSCAT histogram widths (quantified via the coefficient of variation $= \frac{standard\ deviation}{distribution\ mean}$) in the range of 5 – 10 % have been reported for nominally monodisperse samples (e.g. in Young *et al.*[9] Fig. S12c). Assuming the measured samples are truly monodisperse, this width stems from measurement intrinsic and instrument-related errors including e.g. substrate inhomogeneities or focus drifting. The impact of this measurement-related spread on the width of the experimentally obtained iSCAT histograms, however, is strongly dependent on the measured sample. The more heterogeneous the sample is to start with, the less the fluctuations introduced by the measurement matter in terms of the overall distribution width. Since intrinsic and measurement fluctuations combine in quadrature, the coefficient of variation ($CV = \sigma/\mu$) is a robust way to compare relative dispersions across distributions with different means, and the coefficient of variation of the iSCAT contrast should read:

$$CV_{iSCAT} = \sqrt{CV_{sample}^2 + CV_{meas}^2}$$

,where CV_{sample} is the size spread of the sample and $CV_{meas} = \sigma_{meas}/\mu$ is the additional spread due to the uncertainty introduced by the measurement.

If we take the CV_{meas} = 5-10% from Young *et al.* as values for measurement fluctuations introduced by iSCAT and compare the case of a quite monodisperse sample (CV_{sample} = 5 %) with our quite heterogeneous sample ($CV_{TEM} = \frac{2623}{6945} * 100\% \approx 38$ %), we see that the impact of the measurement fluctuations is much higher for the monodisperse sample, while for the heterogeneous sample it has a minor impact on the total CV_{iSCAT}:

Supplementary Table 1. Impact of measurement CV on total CV for monodisperse and heterogeneous samples.

Sample CV (%)	Measurement CV (%)	Total CV (%)
5.0	5.0	$\sqrt{(5^2 + 5^2)} = 7.1$
5.0	10.0	$\sqrt{(5^2 + 10^2)} = 11.2$
38.0	5.0	$\sqrt{(38^2 + 5^2)} = 38.3$
38.0	10.0	$\sqrt{(38^2 + 10^2)} = 39.3$

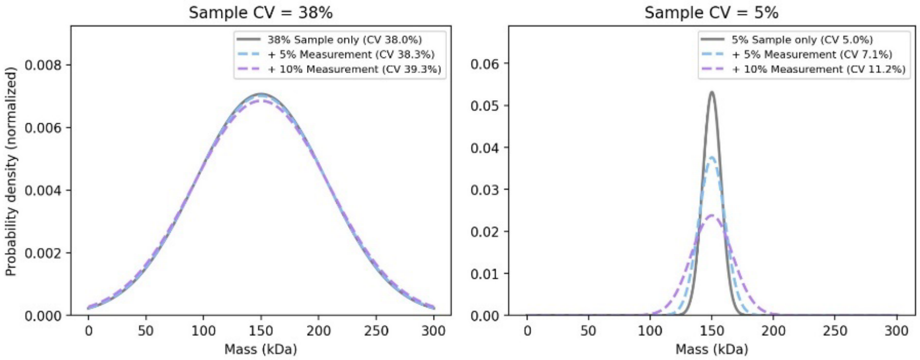

Supplementary Figure 4. Change in distribution width for exemplary monodisperse and heterogeneous samples for different measurement CVs.

The aspects discussed above account for the high agreement of the histograms and CV values obtained from the TEM and iSCAT measurements displayed in Main Text Fig. 1 ($CV_{TEM} = \frac{2623}{6945} \approx 0.38$; $CV_{iSCAT} = \frac{0.385}{1.138} \approx 0.34$). The close agreement is not indicative of higher measurement precision than reported in literature; rather, our measurements are performed on a more heterogeneous sample in which the contribution of measurement noise to the observed histogram width is negligible. In this case, the dominant source of dispersion arises from the intrinsic size heterogeneity of the sample.

Consistent with our findings, recent iSCAT studies on inorganic samples have demonstrated that iSCAT derived size distributions display comparable spreads to those measured by TEM and SEM.[10,11] This further supports the conclusion that, for heterogeneous samples, the observed dispersion is predominantly determined by intrinsic sample heterogeneity rather than by measurement-induced effects.

Calibration fit:

Linearity between iSCAT contrast and particle mass/volume has been reported previously with fits that have R^2 values very close to unity ($R^2 = 0.9997$ in Ref. [12] vs $R^2 = 0.9975$ in our case). Our method differs in that we exploit a single sample's full distribution by matching percentiles of the iSCAT contrast histogram to the corresponding percentiles of the TEM volume distribution; using the entire distribution in this way yields high statistical precision when many particles are available. For our dataset ($n > 1000$) the percentile-fit slope is tightly constrained (fit error $<$ 2%) to a similar precision to what has been obtained by comparing the means of different distributions. As expected, the uncertainty and deviation from linearity decreases when more

particles are included (6.8% for n = 100 to 2.0 % for n= 900), and the linear fit improves accordingly as shown by the figure below where we repeat the analysis with only a subset of the total number of particles.

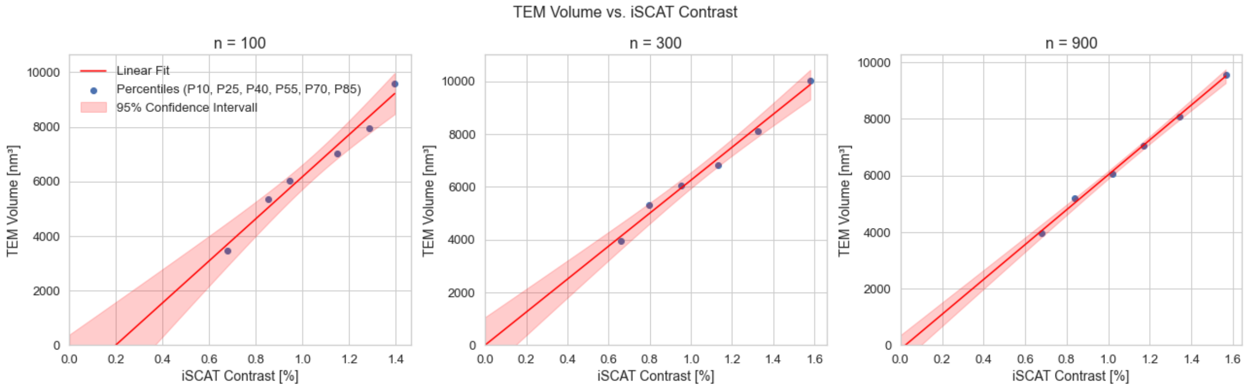

Supplementary Figure 5. Uncertainty of the TEM vs iSCAT calibration fit depending on the size of the dataset, visualized by the 95% confidence band.

From the above discussion and previous iSCAT literature, it is evident that a highly linear relationship with minimal calibration error can be experimentally established between iSCAT contrast and particle volume, provided that a sufficiently large dataset is used. In this case, the uncertainty of the calibration itself becomes negligible. However, this does not imply that each individual particle volume measurement carries such a small uncertainty. Rather, the calibration ensures that no additional error is introduced during the conversion from iSCAT contrast to particle volume.

c) Error estimation

As the PLQY is given by $PLQY = \eta N_{emitted}/N_{absorbed}$ any error in the evaluation of the calibrated correction coefficient η, the number of emitted photons $N_{emitted}$ and the number of $N_{absorbed}$ photons contribute to the uncertainty in the calculated PLQY. In the previous sub-sections, we discussed the major sources of noise in these three terms. As the calculation of PLQY and the calibration of the optical setup involve multiple steps, precisely estimating the overall error is challenging. What we provide here is an estimate to the uncertainty coming from the sources to which we can quantify the error. We can identify the following sources of uncertainty for the number of absorbed photons: iSCAT contrast to particle volume conversion (2 %), iSCAT 638 nm laser bandwidth (0.5 %) and iSCAT contrast fluctuation (~10 % coming from e.g. substrate inhomogeneities, focus drifting). For the iSCAT contrast fluctuations, which have been reported to lie between 5 % and 10 % (see Section 3b), we conservatively use the upper bound of 10 %. Similarly in the number of emitted photons the main sources of error to which we can quantify are: PL excitation laser bandwidth (0.5 %), change of the camera QE efficiency over the PL bandwidth (2 %). As these factors are all multiplicative, the total error is just the quadrature sum of the single relative errors:

$$\sigma_{tot} = \sqrt{\sum_{i}^{N} \sigma_i^2}$$

The combined contributions result in a final error on the PLQY of around 10.4 %, it being dominated by the original fluctuations in the iSCAT contrast estimation. This uncertainty is in the order of error which also bulk measurements typically show.[13] The uncertainty we report here constitutes an estimate on the statistical error on the PLQY and does not consider systematic shifts which are instead included in our correction factor used for calibration against the PLQY obtained from bulk measurements.

d) Dielectric function and possible variations

The dielectric function is an important parameter in the extraction of the single-particle PLQY. Variations in the dielectric function of the measured nanoparticles might also introduce variations to the extracted PLQY and need to be corrected for in the calculation of the absorption cross-section. Therefore, in the following we discuss, in detail, the influences of material properties on the dielectric function in different material systems and specifically in our system of colloidal $CsPbBr_3$ nanocubes.

Variations (between the actual and used dielectric function) may arise either at a systematic level or as particle-to-particle fluctuations:

Systematic shifts:

- **Size dependence**: For very small particles optical constants can change significantly with size, e.g. due to quantum confinement effects[14] or due to plasmonic effects such as Landau damping[15,16]. To account for these effects, correction terms can be included into the equation for determining the dielectric function.[15–17] For instance, Hunger et al. measured the complex polarizability of single gold nanoparticles and showed that the experimentally obtained distribution is in high agreement with the expected distribution when correcting for size effects in the calculation of the dielectric function.[17] Their results also show that the variation in the polarizability/cross sections is caused by the size variation of the measured particles rather than material fluctuation.
  In our case of $CsPbBr_3$ nanocubes with cube length of >12 nm no quantum confinement or plasmonic effects are present, therefore no correction term for the size is needed for the dielectric function.
- **Measurement errors** of the dielectric functions reported in the literature.[14] Such systematic shifts are accounted for in our experimentally determined correction factor.

Particle-to-particle variations:

- **Material composition or structure:** Inhomogeneities in material composition or structure introduced during synthesis can also modify the dielectric function, though the extent of this effect depends strongly on the system. More pronounced influences are expected in cases involving alloying or interface roughness such as in core/shell chalcogenide quantum dots, or compositional gradients in multivalent heterostructures, where ion migration can make the dielectric response dynamic, as observed in complex perovskite systems.
  In contrast, our colloidal $CsPbBr_3$ perovskite nanocrystals are synthesized as single-phase materials without compositional grading or core/shell architectures. Here, local disorder in the form of (surface) vacancies or point defects are present, however we do not expect these to strongly modify the dielectric function. These defects have, however, a significant impact on the PLQY by opening up non-radiative channels,[6,18] resulting in the variability in SP-PLQY reported in Figure 2b of the manuscript. The concept of non-radiative channels having a major impact on the emission but not the extinction has been shown experimentally during blinking events, where the emission changes drastically via activation of non-radiative quenching rates, with negligible influence on the observed extinction cross section.[19]

- **Shape anisotropy** can in principle influence the dielectric function, but even for highly anisotropic shapes (e.g., rods vs. cubes), the effect is only a few percent for $CsPbBr_3$.[20] Also, in a recently published study it was shown that the one-photon absorption (OPA) cross-section of $CsPbBr_3$ nanocrystals with varying dimensionality, including nanocubes, nanoplatelets, and nanorods, scales linearly with nanocrystal volume.[21]

Section 4. Calibrating setup for quantitative emission, excitation and PLQY measurements

a) Setup calibration

For calibrating the setup to collect quantitative emission information we followed a protocol from literature.[22] First, we determined experimentally the collection efficiency of our objective. Due to the anisotropic emission of dipoles near the hexane–coverglass boundary, which is strongly oriented toward the glass, the collection efficiency cannot be simply equated with the solid angle Ω of the objective's numerical aperture. To account for this effect, we covered nanocubes that were dropcasted on a coverglass with immersion oil, resulting in an homogenous surrounding media exhibiting the same refractive index ($n_{coverglass/immersion\ oil} = 1.52$). As there is no preferential direction for the emitting dipoles in the nanocubes, their emission pattern becomes isotropic. In this case, the collection efficiency of the objectives simply corresponds to the solid angle Ω covered by its numerical aperture:

$$I_{oil} = \frac{4\pi}{\Omega} I_{tot}$$

Where I_{oil} is the detected PL intensity with the oil on top and I_{tot} is the total emitted intensity from the nanocubes. The collected intensity in the real sample I_{smpl} is related to the total emitted intensity by:

$$I_{smpl} = \eta I_{tot}$$

By using the intensity collected with the oil on top, the collection efficiency in the real-sample conditions can be extracted by:

$$\eta = \frac{I_{tot}}{I_{smpl}} = \frac{\Omega}{4\pi} \cdot \frac{I_{oil}}{I_{smpl}}$$

Knowing the solid collection angle, and after measuring the emission intensity of the sample with oil on top I_{oil} and the real sample without oil at the same excitation conditions I_{smpl}, one can calculate the collection efficiency η of our objective for the real sample under measurement conditions. For the combination of our objective (Olympus, 100x, NA = 1.42) and our sample we get a collection efficiency of 74.6 %.

Next, we determined the transmission losses of the setup in the excitation and detection path due to the optical elements (such as the 50/50 beamsplitter) in the beam path.[22] For the detection path, we determined the actual laser power arriving at the sample plane without the objective. For this, we determined the laser power lost in the excitation channel (λ = 450 nm) by measuring the power after the incoupling fiber and at the sample plane after removing the objective (0.465 transmitted). Afterwards, we introduced a powermeter with a beamsplitter into the beam path and calibrated its recorded measurements to reflect the actual laser power at the objective position. Therefore, this value did not need to be included again in the correction factor applied in the following analysis. The transmission loss of the objective was determined separately by checking the power before

and after the objective (0.83 transmitted) – this value needs to be included twice in the correction factor as both the excitation and the emission are affected by it.

Next, we investigated how much of the emitted photons are lost inside the detection path. To account for the losses in the detection channel, we measured first the laser power at the position of the removed objective, then placed a mirror at this position and measured the laser power at the camera position (0.30 transmitted, including the effects of the used filters and dichroic mirrors). The total loss factor (excluding the excitation losses which were included already in the documented laser power) amounts to 0.156. To correct for the underestimated detected photons and the overestimated excitation, the PLQY has to be multiplied in the python script by a correction factor of 6.42 (= 1/0.156). Additionally, included in the python script is the correction factor obtained from the bulk reference measurements (*1.18; described in main text and Supplementary Figure 15) and for the quantum efficiency of our fluorescence detection camera (*1.09 = 1/0.92).

b) Laser power excitation in the linear regime

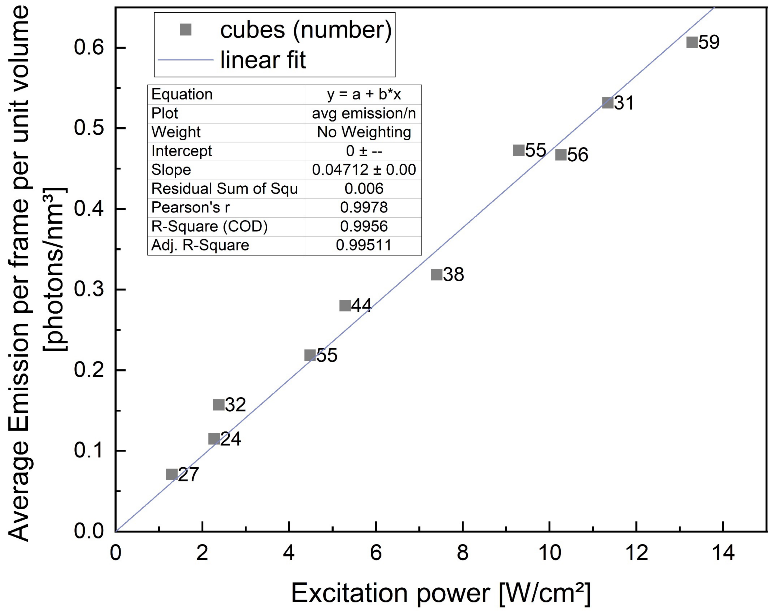

Supplementary Figure 6. Excitation power dependence (λ = 450 nm) measured on single cubes with our optical setup, showing a linear trend of emission increase (per unit volume; volume determined by iSCAT) with increasing excitation power. This reference measurement confirms that our typical excitation range (4 and 8 W/cm²; see methods) only exciton recombination occurs, without higher-order processes such as Auger recombination or exciton-exciton annihilation.

Section 5. Python script analysis and workflow

In our experiments, we simultaneously capture the iSCAT and PL signals from perovskite nanocubes attaching to the coverglass using two separate cameras. To determine the PLQY of multiple nanoparticles, we developed a custom algorithm for particle localization and identification, enabling the accurate pairing of iSCAT signals with their corresponding PL emissions.

Initially, iSCAT images undergo background correction using DRA and are analyzed with the PiSCAT module (see Methods for details). This analysis provides the localization coordinates, contrast, and attachment frame for each particle. A summary of this process is presented in Supplementary Figure 7a, where each localized particle in the whole image stack is marked by a white square. Particles that detach shortly after landing are excluded from further analysis and are marked with red squares. In the DRA images, detaching particles are readily identified by their positive (white) contrast, which is the inverse of the signature associated with particle landing on the substrate.

As a result, we generate a histogram of particle contrast values (Supplementary Figure 7b), where the lowest bin includes all excluded particles, along with their spatial distribution within the camera frame.

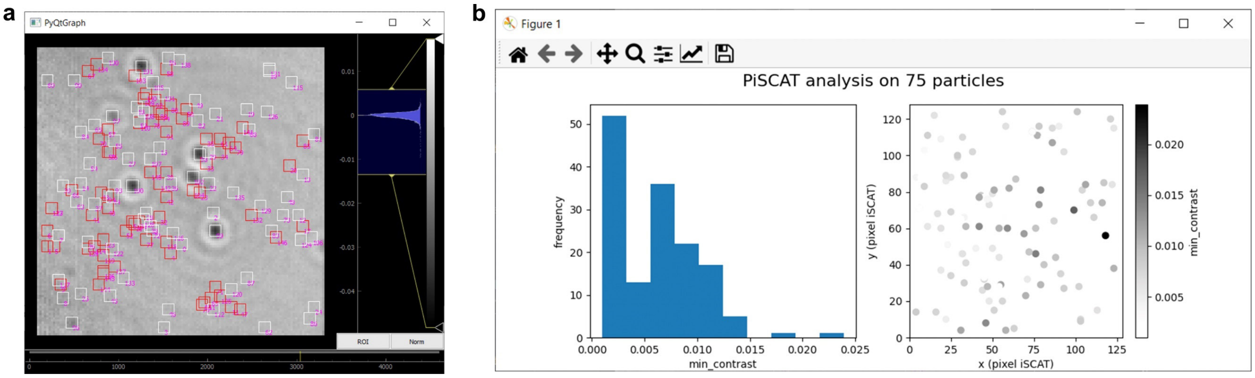

Supplementary Figure 7. Localization of attaching nanocubes through PiSCAT. **a** Localized particles in the image stack are indicated by white squares. Particles that detach soon after landing are indicated by red squares and are not considered for the following analysis. **b** Histogram and spatial distribution of detected particles.

Prior to comparison with the iSCAT data, PL images are rescaled and binned to ensure that the number of pixels in both image sets aligns. The pixel values in the PL images are then converted from grayscale counts to the actual number of photons detected. This conversion involves subtracting a baseline corresponding to dark counts and accounting for the camera's quantum efficiency at 515 nm (see Ref. [23] for more details and methods section).

For accurate comparison between the iSCAT and PL data, it is essential to account for differences in image normalization. While iSCAT images are normalized, making the detected contrast independent of the incident laser power, raw PL images are not, as the emission is proportional to

the number of absorbed photons. Due to the Gaussian profile of the illumination beam, particles at the edges of the field of view exhibit lower PL emission than those at the center.

To address this, the non-normalized iSCAT background, which represents the illumination beam's intensity profile, is used. This background is scaled to a maximum value of 1 and blurred to remove local variations. The PL images are then divided by this adjusted background, effectively normalizing the particle signals so that those near the edges of the field of view are scaled as if they were at the center. With these corrected PL images, the comparison with the iSCAT data is performed.

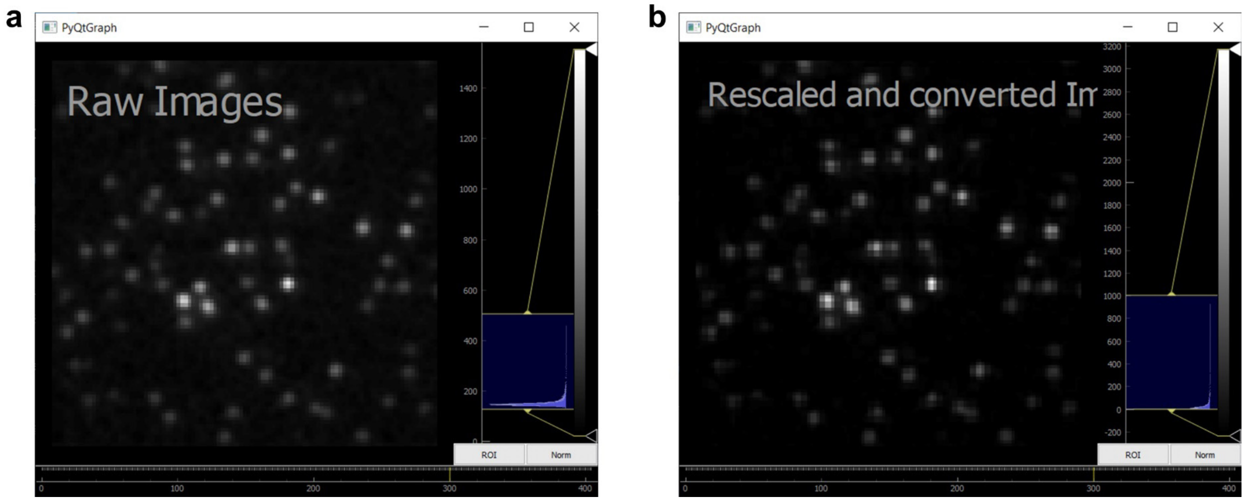

Supplementary Figure 8. Rescaling raw PL images. Raw images (**a**) are rescaled and binned (**b**) so that the number of pixel is equal to the iSCAT data, and converted to emitted photons instead of gray-levels.

Although experimental efforts can be made to perfectly align iSCAT and PL images, temporal misalignment makes it impractical to directly map particle positions in the PL images based on those inferred from the iSCAT data. Consequently, some displacement in both the x-y coordinates and the frame of arrival is expected between the iSCAT and PL videos.

To address this, for each particle localized in the iSCAT analysis, we search for a corresponding particle in the PL dataset by restricting the search to a specific spatial and temporal region around the position and timing identified in the iSCAT analysis. Within these confined PL regions, particle localization is performed again using the *trackpy* module (see Supplementary Figure 9).[24]

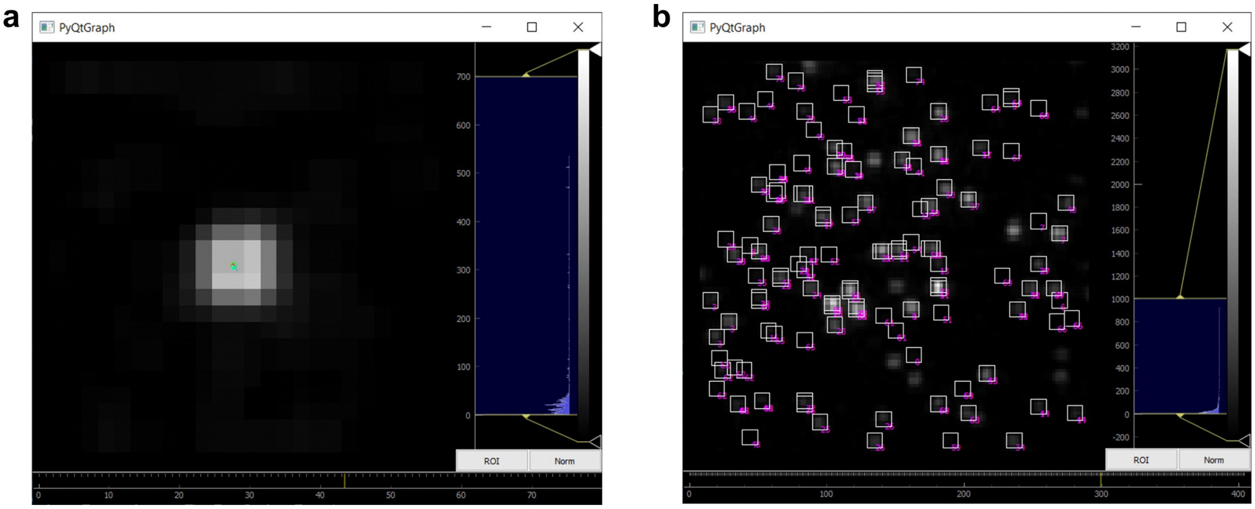

Supplementary Figure 9. Single particle localization is run again in the PL videos. Instead of running the localization on the whole video, for each particle detected in the iSCAT analysis we cut a small PL video (**a**, centered both in space and time around the iSCAT localization) and run the analysis in this sub-dataset. **b** Overview of localized particles.

If only a single particle is identified in the PL subset, it is directly associated with the corresponding particle from the iSCAT data. However, special attention is required when multiple particles are detected in the PL localization. A key difference between the iSCAT and PL datasets lies in their signal behavior: in iSCAT, the signal appears only during particle attachment or detachment due to DRA background subtraction, whereas in PL, the signal is continuous and only vanishes when the particle detaches.

In cases where multiple particles are present in the PL video, the correct particle is identified based on its attachment within the selected sub-dataset. PL intensity traces are collected across the frames, smoothed using a rolling average, and analyzed via linear fitting (see Supplementary Figure 10). A particle that attaches during the frames of interest exhibits a steep slope in the linear fit (indicating a transition from no signal to a high signal), whereas particles present throughout the sub-dataset display much lower slopes. The correct particle, identified in Supplementary Figure 10 by a small green circle, is selected as the one with the highest slope in the linear fit, corresponding to the particle detected in the iSCAT analysis.

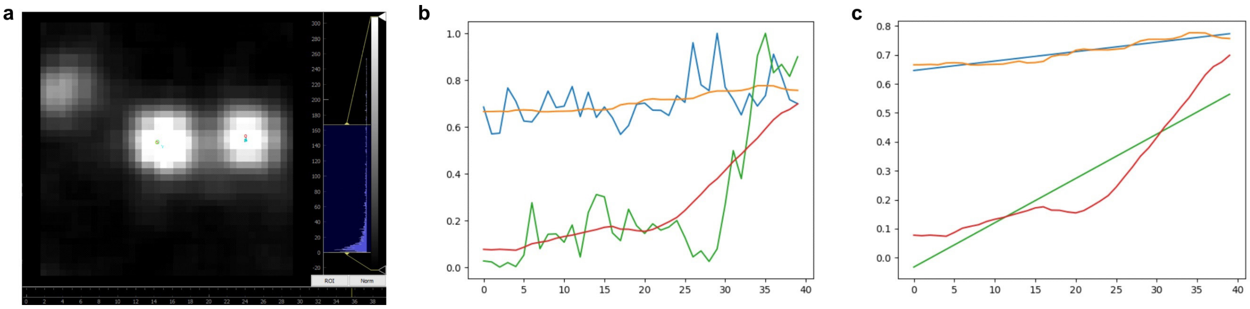

Supplementary Figure 10. Determination of the right particle when more than one is found by the localization applied to the PL images (**a**). We take the time-trace of the signal and smooth it with a rolling average (**b**). We then perform a linear fit (**c**). The

particle with the highest linear coefficient is deemed to be the one detected by the iSCAT analysis, because it corresponds to a particle that has recently appeared in the video.

Applying this procedure to each iSCAT particle generates a map of overlapping localization points from the iSCAT and PL images (see Supplementary Figure 11a, with black numbers indicating iSCAT positions and red numbers indicating PL positions). This mapping allows the identification and exclusion of “false positive” particles that do not correspond between the iSCAT and PL datasets.

By calculating the mean distance between the iSCAT and PL localizations, we estimate the spatial shift between the two image sets. Particles for which the distance between iSCAT and PL localizations exceeds a threshold based on the mean displacement are excluded from further analysis (see Supplementary Figure 11b).

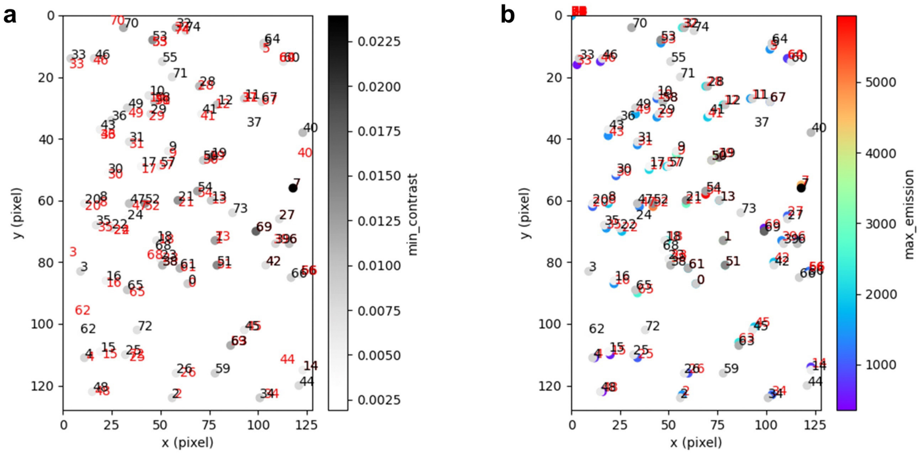

Supplementary Figure 11. a Map of the iSCAT and PL localizations found from the analysis. Localizations from iSCAT are highlighted by black numbers, while the ones from the PL images are shown in red. From all the localizations, a mean displacement between the two images is calculated, and corrected for (**b**). All particles for which the iSCAT and PL distance is still above a certain threshold are excluded (black numbers with no corresponding red ones assigned).

Additionally, we exclude cases that could compromise an accurate evaluation of the PLQY. For instance, particles with overlapping PL emissions are excluded, as this overlap could lead to an overestimation of their PL signal. Similarly, particles that exhibit PL signals from the start of the dataset or those with abruptly terminating PL signals - potentially indicating detachment from the coverglass - are also excluded.

After applying these additional filters, only a subset of particles remains, represented by overlapping green circles and red squares in the example shown in Supplementary Figure 12. Furthermore, particles landing near the end of the dataset are excluded, as their limited frame count does not allow for the extraction of meaningful PL traces.

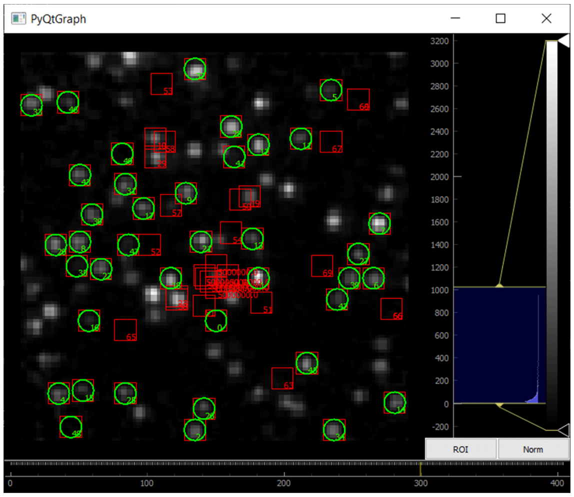

Supplementary Figure 12. Particles for which the PL PSF overlaps, are in the video from its beginning or of which their PL disappears completely during the dataset are excluded. This leaves for further analysis only particles highlighted by overlapping green circles and red squares.

For each remaining particle, the PL signal is extracted by integrating the emission within the green circles shown in Supplementary Figure 12. This enables the determination of several key PL parameters, such as maximum emission intensity and blinking traces (see Supplementary Figure 13). As demonstrated in the main text, iSCAT contrast values can be converted into nanocube volumes, which are then used to calculate the PLQY at the single-particle level.

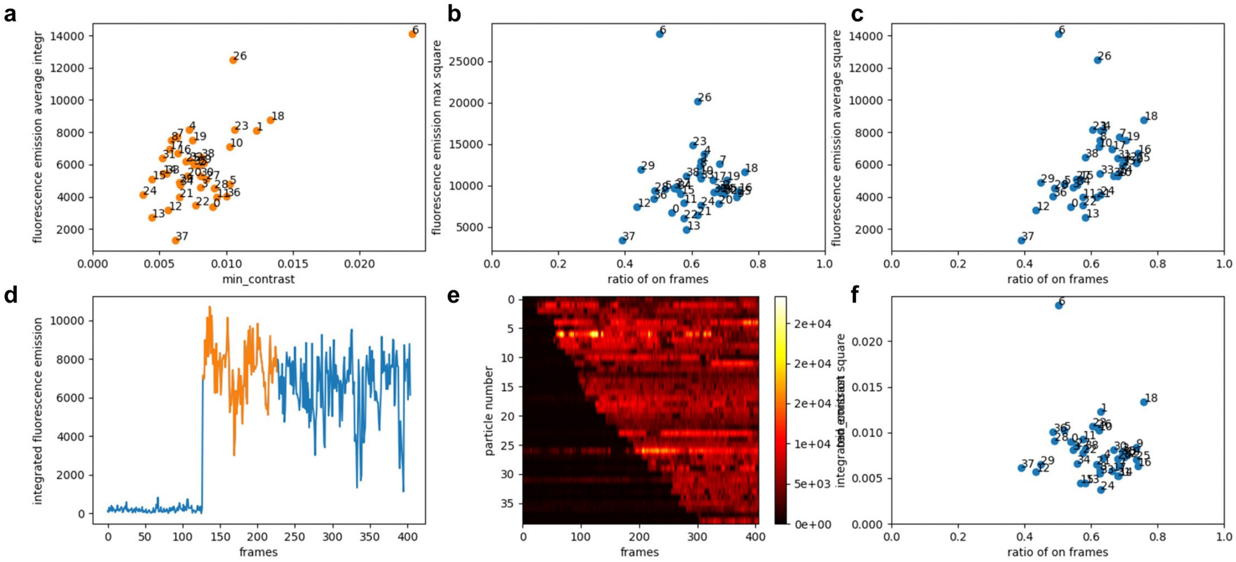

Supplementary Figure 13. Summary of information extracted from the correlated iSCAT and PL analysis. **a** iSCAT contrast vs PL emission averaged over a number of frames. **b,c** Squared maximum and average emission over PL traces vs. ratio of on to off frames (defined as frames with emission below a certain threshold calculated from the maximum emission of each particle). **d** Example of a PL trace, the orange part indicates the part of the trace used to calculate PL properties. For a fair comparison, we keep the number of frames used to be the same of each particle. **e** PL traces for all the particles detected in this particular measurement run. **f** Ratio of on images plotted against the corresponding iSCAT contrast.

Section 6. PLQY reference bulk

In this section, we compare traditional bulk PLQY measurement techniques with our all-optical iSCAT-based approach.
PLQY measurements of diluted samples are challenging with these traditional techniques due to their detection thresholds. For this reason, we used 2.5 times the typical sample volume (25 µL instead of 10 µL; undiluted instead of 1:100 diluted) in this set of measurements compared to standard iSCAT experiments. The cube sample used was redispersed in 100% enhancement solution post-synthesis. The enhancement solution provided adequate ligands and $PbBr_2$ salt, ensuring that dilution had no impact on the quantum yield (QY). This was validated through the dilution experiments shown below.

Reference method – Fluorescein dye:

The PLQY of $CsPbBr_3$ colloidal solutions in hexane at lower concentrations than the usual concentrations used in bulk measurements (< 0.005 OD), comparable to iSCAT measurements, was determined with the comparative method, using a solution of fluorescein in 0.1M NaOH as a reference dye. A fresh stock solution (1 mM) in 0.1M NaOH was prepared immediately before use and further diluted to prepare 10 reference samples with different concentrations below 10 µM to avoid interference of concentration effects. Similarly, a colloidal solution of $CsPbBr_3$ NCs was diluted with n-hexane to obtain 10 samples with different concentrations (0.0002 < OD < 0.005). Absorbance and photoluminescence spectra of all references and samples were measured on a commercial spectrometer (*FluoroMax-4Plus,* equipped with an F-3031 transmission accessory, *HORIBA Scientific*) at identical slit width. The excitation wavelength for PL spectra was set to 450 nm, and each sample was measured twice, including additional background measurements of n-hexane and 0.1M NaOH.
The baseline of all absorbance spectra is corrected by subtraction of the minimum value, so that the optical density @540 nm = 0. The baseline offset required for correction is set as the respective error value. PL spectra are corrected by subtraction of the PL from blank (solvent) samples and then integrated over the full measurement range (470-700 nm). The corrected average integrated PL intensity and respective standard deviation is calculated from the two consecutive PL measurements of each sample. It was then plotted against the optical density at the excitation wavelength (450 nm) and fitted with a linear function as given below, for which the y-intercept $b = 0$.

$$y(x) = m_i \cdot x + b$$

The slope, or gradient m_i is derived from the linear fit function.

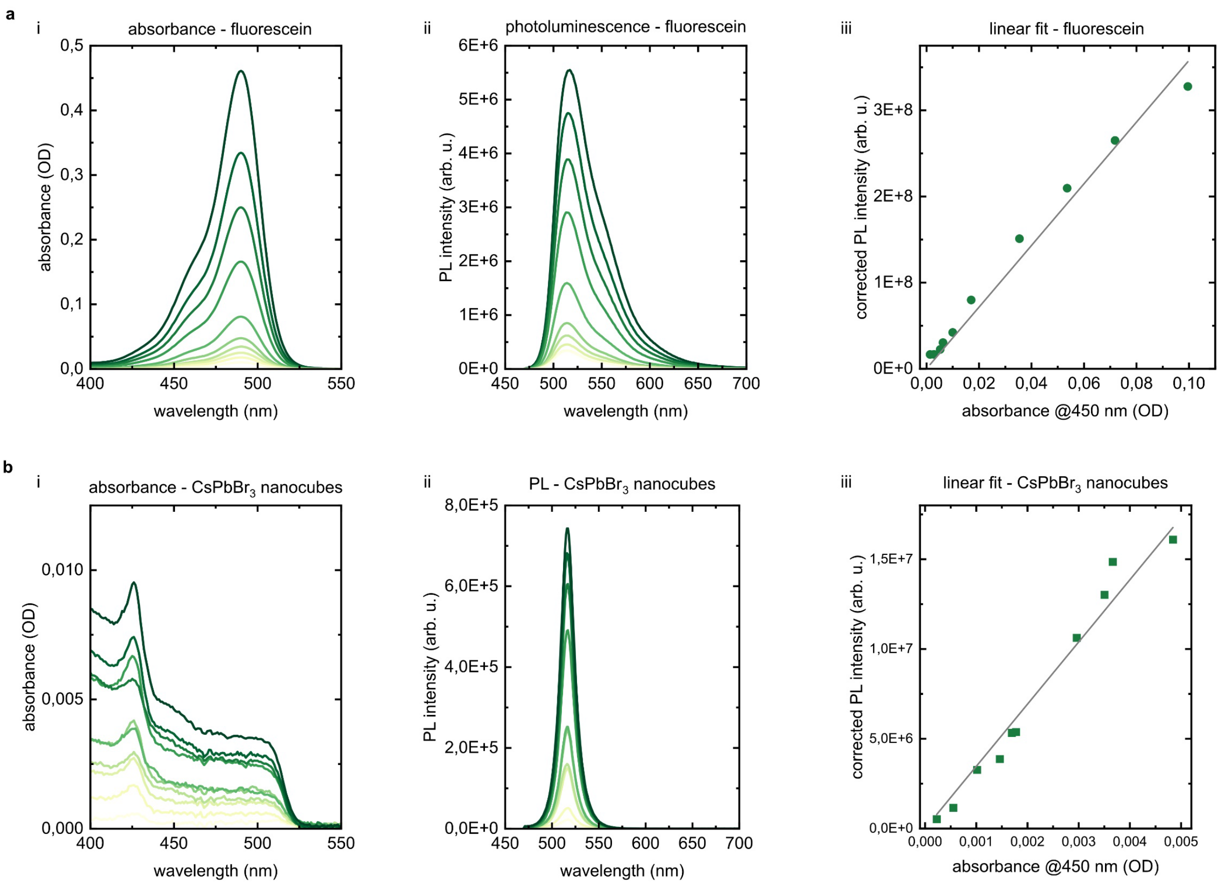

Supplementary Figure 14. Determining PLQY of the $CsPbBr_3$ nanocubes by fluorescein dye reference measurements. **a** Fluorescein measurements of solutions with different dye concentrations. i, Absorbance; ii, Photoluminescence; iii, Linear fit (m_R = 3.525E9 ± 1.202E8, R^2 = 0.99) of PL and absorbance. (x,y) error bars not included in plots but considered for linear fits. **b** corresponding $CsPbBr_3$ measurements (m_{NC} = 3.579E9 ± 1.175E8, R^2 = 0.99). The sharp absorption peak observed around 425 nm can be attributed to a minor side product of thin $CsPbBr_3$ nanoplatelets. However, setting the excitation wavelength to 450 nm excludes the possibility of interference with the measurements for PLQY determination.

Finally, the PLQY of $CsPbBr_3$ nanocrystals Φ_{NC} dispersed in n-hexane ($n_{NC} = 1.375$)[25] was then calculated as follows from the PLQY of the fluorescein reference dye ($\Phi_R = 0.79$)[26] in 0.1M NaOH ($n_R = 1.335$)[27] and the respective values of m_{NC}, m_R.

$$\Phi_{NC} = \Phi_{\mathrm{R}} \cdot \left(\frac{m_{NC}}{m_R}\right)\left(\frac{n_{NC}^2}{n_R^2}\right)$$

An error value $\Delta\Phi_{NC}$ is calculated according to Gauss' law of error propagation.

$$\Delta\Phi_{NC} = \pm\sqrt{\left(\left(\frac{\partial\Phi_{\mathrm{NC}}}{\partial\Phi_{\mathrm{R}}}\right)\cdot\Delta\Phi_R\right)^2 + \left(\left(\frac{\partial\Phi_{\mathrm{NC}}}{\partial m_{NC}}\right)\cdot\Delta m_{NC}\right)^2 + \left(\left(\frac{\partial\Phi_{\mathrm{NC}}}{\partial m_R}\right)\cdot\Delta m_R\right)^2}$$

This yielded $PLQY_{\text{fluorescein reference}}$ = 83 ± 6 %.

Direct method – Integrating sphere:

By using the integrating sphere method (as described in method section), we arrive at $PLQY_{integrating\ sphere}$ = 84 ± 5 %. The value is corrected for indirect excitation by scattered photons following a method by de Mello et al.[28]

iSCAT approach – Ensemble PLQY based on single particle information:

By sample-averaging the single particle results from iSCAT measurements we arrive at $PLQY_{iSCAT}$ = 71 ± 3 %.

To improve accuracy, we used a volume-weighted calculation rather than a simply averaging the PLQY of all cubes, as QY can vary with particle volume. Smaller particles might influence the average disproportionately due to this, whereas larger particles, with their higher absolute absorption and emission, play a more dominant role for the absolute ensemble QY. The volume-weighted PLQY was calculated by taking the QY of each particle, multiplying it by its volume fraction (particle volume divided by the total volume of all particles), and summing the contributions. This method resulted in a weighted PLQY of 71%, slightly lower than the simple average of 73%.

Comparison of results:

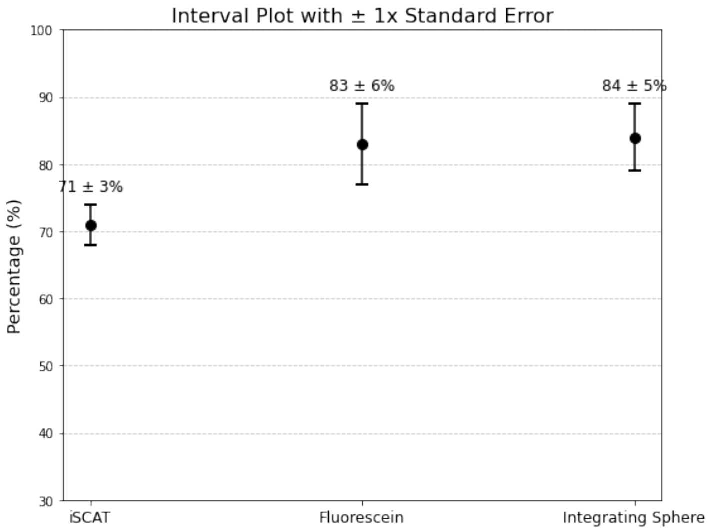

Supplementary Figure 15. Comparison of iSCAT ensemble-averaged value with values obtained by established bulk methods (i.e. fluorescein dye reference and integrating sphere) measured on the same day, sample and concentration. Interval plots with ±1 standard error (SE) show good agreement, with iSCAT slightly underestimating the PLQY. We attribute the observed difference in PLQY to several factors: the approximation of a homogeneous particle environment in the absorption cross-section calculations, deviations in the perovskite´s dielectric function from literature values, and calibration uncertainties in the optical setup’s collection efficiency. This experimentally determined offset (83.5/71 = 1.18) is incorporated as a reference factor in subsequent measurements to account for these effects.

Section 7. Bulk treatments with enhancement solution and oleylamine

Colloidal solutions of as-synthesized $CsPbBr_3$ nanocubes were treated with

i) oleylamine

ii) enhancement solution, containing oleylamine, oleic acid and $PbBr_2$

to study how their addition affects the optical and morphological properties of $CsPbBr_3$ nanocubes. For these experiments, aliquots of $CsPbBr_3$ NCs dispersed in n-hexane were diluted and mixed with different volumes of oleylamine or enhancement solution, as listed in Supplementary Table 2. Samples were stirred at room temperature for 30-60 minutes, followed by optical characterization on the same day.

Supplementary Table 2. Post-synthesis treatment of colloidal $CsPbBr_3$ nanocubes with oleylamine (OAm) or enhancement solution, volumes V given in µL. PLQY values were measured in an integrated sphere on the same day. Sample 6 was non-emissive after treatment with oleylamine, and no PLQY could be determined.

**sample no.**	***V*(CsPbBr3)**	***V* (OAm)**	***V*(enhance)**	***V* (n-hexane)**	PLQY (%)
**0**	10	0	0	90	55.54 ± 1.958
**1**	10	0.08	0	80	53.61 ± 1.489
**2**	10	0.33	0	60	61.10 ± 1.671
**3**	10	0.83	0	10	59.30 ± 1.571
**4**	10	1.67	0	90	71.60 ± 2.061
**5**	10	6.67	0	90	64.92 ± 2.066
**6**	10	16.67	0	80	-
**7**	10	0	8.33	80	86.92 ± 1.848
**8**	10	0	33.33	60	95.66 ± 2.208
**9**	10	0	83.33	10	93.94 ± 2.181

All samples treated with OAm lost their emission properties after two days, indicating that this post-synthesis enhancement is unsuitable for long-term stability.

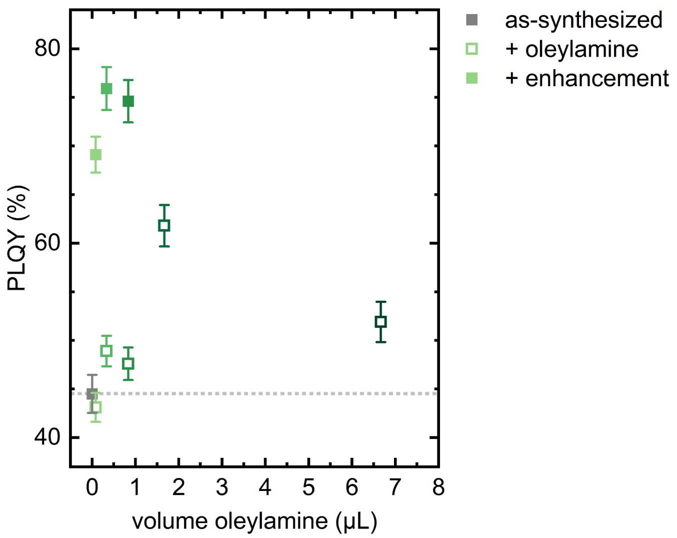

Supplementary Figure 16. Bulk enhancement measurements. Scatter plot of PLQY as a function of the volume of oleylamine added to the sample (in case of enhancement solution, OAm constitutes 1% of the added mixture; see also Supplementary Table 2). The addition of OAm alone initially increases PLQY, followed by a decrease with further addition. The addition of enhancement solution leads to a greater PLQY increase compared to OAm alone. Samples treated with OAm alone showed no measurable emission two days post-treatment, whereas those treated with the enhancement solution maintained stable PLQY values. Differences in the absolute values to single particle measurements shown in Fig. 3 are attributed to different starting conditions of the colloidal samples, with the bulk measurements conducted 7 months later and at a substantially lower dilution (1:10).

Section 8. Relative PLQY increase per OAm addition step

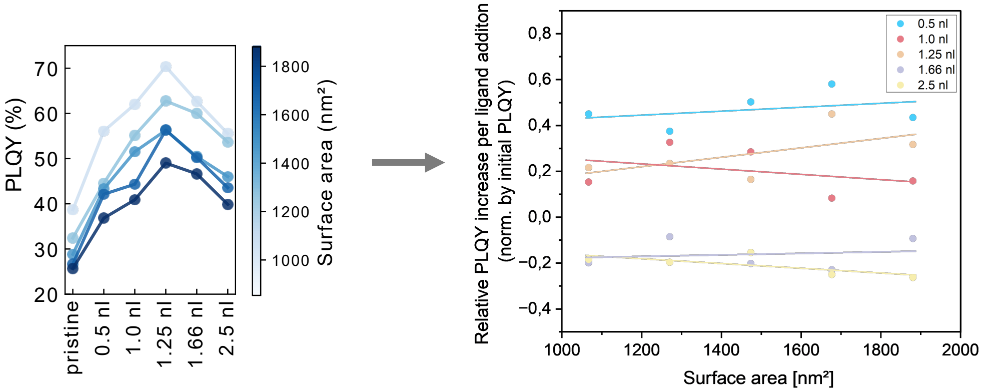

Supplementary Figure 17. Left, absolute change in PLQY versus OAm ligand addition step, with crystals being grouped into surface area subsets. Right, relative change in PLQY (normalized by starting value) versus surface area grouped into ligand addition steps. Each line represents a ligand addition event. For each event, the relative increase in PLQY is plotted versus the surface area.

Section 9. Mechanism of OAm enhancement

Since single-particle measurements in liquids remain challenging, few studies have addressed ligand effects. Among those available, the prevailing assumption is that adding OAm - known to enhance quantum yield in ensemble measurements - enhances PLQY primarily by extending nanocrystal ON-times or increasing the fraction of emissive cubes.[29–31] This discussion highlights a key challenge in the field: true mechanistic insight requires direct quantification of quantum yield (QY) at the single-particle level, as only this allows emission parameters - such as maximum emission - to be placed in a quantitative context and the presence of dark particles to be verified. Using iSCAT, which detects scattering from all particles,[19] no dark nanocubes were observed, and we show that single-particle QY enhancements mirror the trend observed in bulk (Fig. 3 and Supplementary Figure 16), demonstrating that the improvement arises from intrinsic changes within individual cubes rather than from an increasing fraction of emissive ones. Our data further reveals that OAm primarily increases the maximum emission intensity (Fig. 3e), not the ON-time probability as previously assumed, consistent with passivation of shallow surface traps or undercoordinated sites. In contrast, the ON-time fraction is predominantly governed by halide vacancies (Fig. 3d), which create deep trap states and can be mitigated by halide replenishment via $PbBr_2$, which aligns with insights from literature[32]. Thus, while both ON percentage and maximum emission are functionally linked to PLQY - and both improve upon post-synthetic treatment - they contribute to the enhancement through distinct mechanistic pathways involving different classes of defects. Their relative importance may vary with ligand and crystal chemistry - for example, tighter-binding ligands might more strongly affect ON-time[30].

Section 10. Anticorrelation of PLQY and size in pristine samples

Expectation:

In a model system, the PLQY is defined as the ratio of the radiative decay rate to the total decay rate (the sum of radiative and internal nonradiative decay channels + additional nonradiative channels related to a lossy environment - which can be neglected here, as all the materials surrounding the particles have negligible absorption):[33]

$$PLQY\ (a) = \frac{k_r}{k_r + k_{nr}}$$

Which, in its simplest assumption, has **no size dependence** and is only a function of the intrinsic particle properties and of the surrounding environment.

Observation:

In contrast, the **experimental data show a clear size dependence**: larger nanocubes exhibit lower PLQY values.

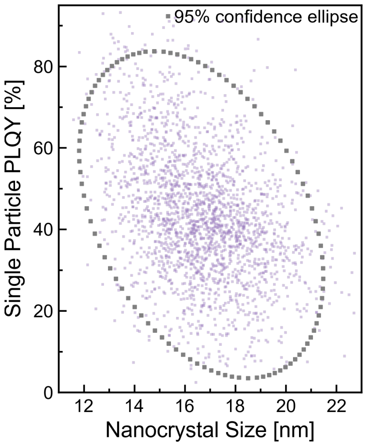

Fig. 2d, ii Scatter plot of PLQY versus nanocrystal size of a pristine $CsPbBr_3$ sample (n=2224).

To better quantitatively assess the scaling, we compress our extensive dataset (n > 2000) into several subpopulations with bin size of 1 nm (Supplementary Figure 18).

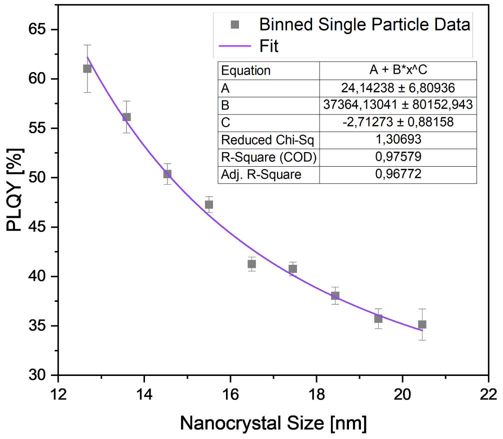

Supplementary Figure 18. Data set of Main Fig. 2d, ii grouped into bins of equal width (1 nm; n > 2000). A phenomenological model is used to fit the data.

We propose a simple phenomenological model (as simple as possible to not overfit) to fit the data in the measured size range: $PLQY = PLQY_0 + k * a^b$ (where $PLQY_0$ is a phenomenological baseline, k is a coefficient, a is the cube side length and b is the scaling). Using this fit, we find an effective scaling behaviour of $\propto a^{-2.7}$, indicating that the dependency (2.7) lies between a surface (2) and a volume effect (3). It should be noted that, while we can fit the scaling of the PLQY from the experimental data, relating it to the scaling of the individual nonradiative channels is not trivial as the relation is not simply $PLQY = 1/k_{nr}$ (due to the presence of the radiative decay rate k_r) and includes both volume and surface contributions, of which we can only observe the combined effect. It must also be noted that the simple model we employ here can be used to fit the size-scaling only in the limited size range we are investigating, as the proposed formula for the PLQY diverges for vanishingly small cubes for b < 0.

Interpretation:

Type of defect correlated with the PLQY offset:

From the in situ measurements (Fig. 3, Extended Data Fig. 2) we know that:

- The initial difference in PLQY is not due to unpassivated sites, as the PLQY offset between large and small cubes persists across all OAm ligand addition steps - including at full coverage (1.25 nL) - and thus cannot be healed by ligand passivation alone (see Extended Data Fig. 2b)
- The difference needs to be correlated to halide vacancies, as the addition of $PbBr_2$ + ligands can level the PLQY difference (see Extended Data Fig. 2a)

➔ We therefore attribute the size dependence of the PLQY in the pristine sample to the effect of halide vacancies.

Scaling of the dependency:

The presence of defects in the bulk and surface adds additional nonradiative channels, leading to a more general expression of the PLQY:

$$PLQY\ (a) = \frac{k_r}{k_r + k_{nr}^{intr} + k_{nr}^{bulk}(a) + k_{nr}^{surf}(a)}$$

Where k_{nr}^{intr} is the intrinsic nonradiative decay in the absence of defects, to which the contributions from nonradiative channel stemming from bulk and surface defects are added. Nanocubes in the size region that we used are expected to have almost no bulk defects and this type of defect is therefore not considered in the following.

In a simplified picture, one can think of the way in which the number of surface defects affects the scaling of nonradiative decay (and thus the PLQY) as having two distinct limits:

- In very small cubes, comparable to the exciton size (a ~ 7 nm = exciton Bohr radius), e-h pairs (either bound as excitons or free charge carriers) can interact with essentially all defects present in the crystal. In this regime, the absolute number of surface defects per particle is what matters; accordingly, the nonradiative decay rate should scale with surface area $k_{nr} = k_{nr}^{intr} + \alpha a^2$, leading to a PLQY that follows an inverse-square ($1/a^2$) dependence.

- On the opposite hand, in very big cubes (a >> Bohr radius), e-h pairs "see" only a small part of the whole particle, meaning that their decay is now determined by the density of defects, and not by their absolute number. Assuming the surface density of defects to be constant, we thus expect the PLQY of large cubes to converge to a constant value, $PLQY_0$, simply because of volume-to-surface ratio considerations: $k_{nr}^{surf}(a) \propto \frac{A}{V} \sim \frac{1}{a}$. We therefore expect the nonradiative decay to behave as $k_{nr} = k_{nr}^{intr} + \beta \frac{1}{a}$ tending to a constant value for big a. In even larger cubes, bulk defects will emerge as an additional factor.

Given the size of the investigated nanocubes and the observed PLQY-size scaling, we conclude that our sample corresponds more closely to the first regime, in which **the absolute number of defects, rather than their density,** determines the behavior.

However, in our experiments we observe a scaling of PLQY to particle size that decreases faster than $1/a^2$ (we fit with an exponent 2.7). To account for this behavior, we need to

examine in more detail the types of e-h pairs present in the nanocubes and responsible for the PL emission:

The Saha equation describes the equilibrium between the creation of excitons or free charge carriers inside of the crystals after excitation:[34,35]

$$\frac{x^2}{1-x} = \frac{1}{n}\left(\frac{2\pi\mu k_B T}{h^2}\right)^{3/2} e^{-E_B/(k_B T)}$$

- For small cubes, excitons are favored, which are less susceptible to interactions with shallow defects due to their high exciton binding energy, resulting in a higher PLQY.
- For big cubes, unbound e-h pairs become more common, which are more prone to couple to defects, resulting in a lower PLQY.

Since in the linear excitation regime each crystal contains at most one electron–hole pair, the carrier density, and thus the Saha effect, scales inversely with volume, introducing a volume dependence in the PLQY that favors smaller cubes. For very big crystals, this effect is expected to be less important.

These effects naturally produce intermediate scaling laws and effective power-law exponents between the small- and large-particle limits, surface and volume effects, which is what we see here with our 2.7 scaling. We believe future work in this direction to be compelling and enabled by our high-throughput single-particle framework.

Section 11. Degradation of single cubes traced simultaneously by iSCAT and PL

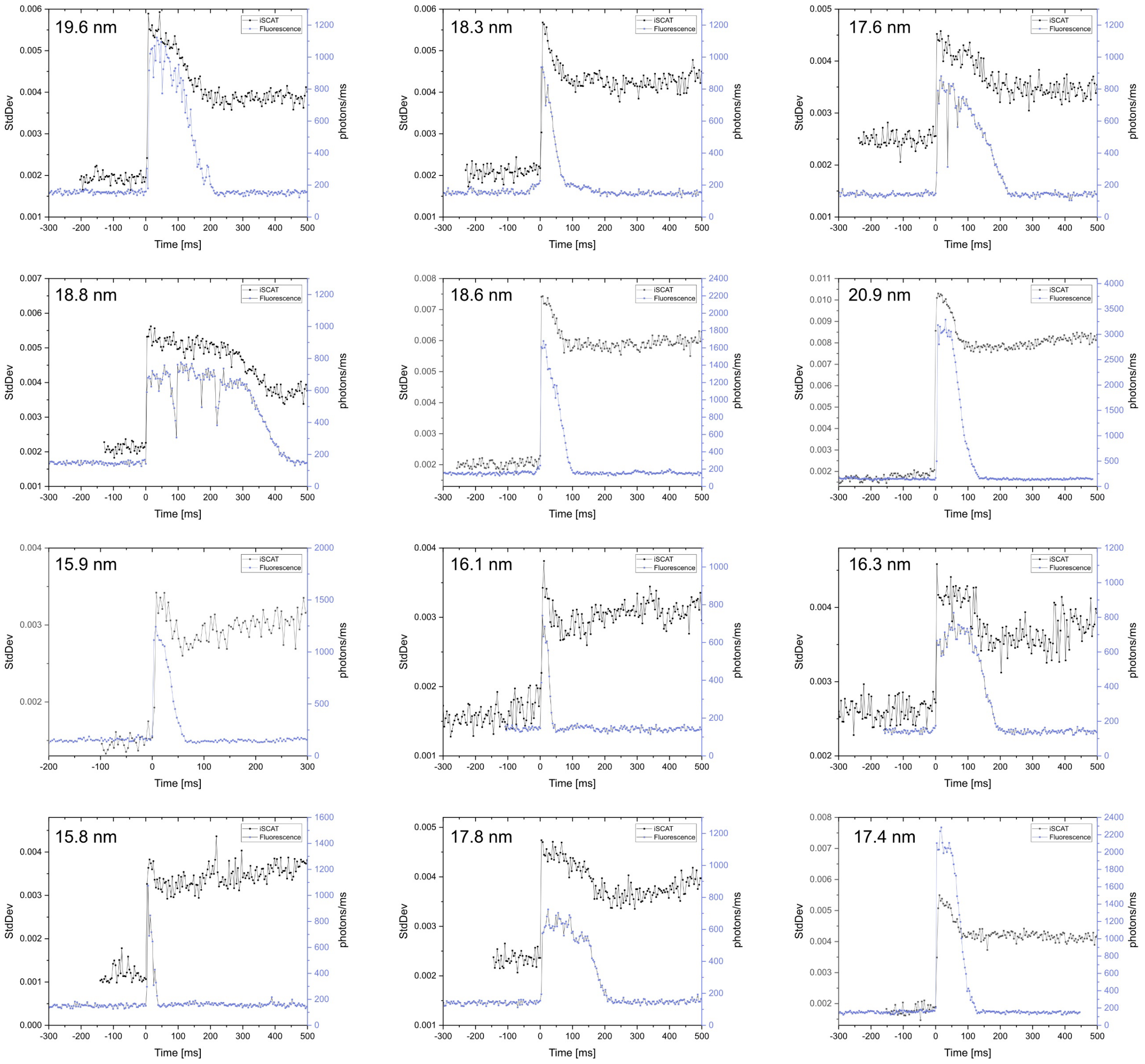

Supplementary Figure 19. Attachment and photoinduced degradation (λ = 450 nm) of single perovskite nanocubes traced by iSCAT (grey lines) and PL microscopy (blue lines). The change in iSCAT contrast is visualized by plotting the standard deviation (StdDev) of the pixel intensity values for each particle region (10 x 10 pixels around the particle center) in the processed iSCAT image. The PL intensities were determined by integrating the pixel values in the corresponding PL image regions. The initial size of the cubes was determined by converting the initial iSCAT contrast upon attachment to size (contrast-to-volume formula at wavelength 450 nm was obtained from simulations, see Section 13). For all cubes, iSCAT and PL signal increases drastically upon cube attachment, followed by a decrease in iSCAT signal and a complete loss of PL. The iSCAT signal decrease occurs over the same timeframe as the PL decrease for all particles.

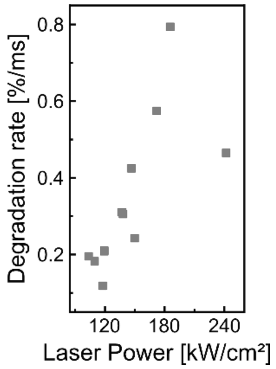

Supplementary Figure 20. Scatter plot of the iSCAT degradation rate (defined as percentage of volume lost per ms) of the individual nanocubes versus the laser power.

Section 12. SEM and XPS measurements on sample with illuminated and pristine cube region

To gain insights into the degradation process, we illuminated a large area of the substrate until several hundreds of nanocubes had attached.

a) SEM

We then imaged the substrate under an SEM, comparing an illuminated with a non-illuminated region (Supplementary Figure 21). Notably, we found that the particles in both regions were cubic in shape, with the nanocubes in the illuminated region having 2.3 nm shorter side lengths, equivalent to a 27% volume loss on average (Fig. 4e). This size reduction matches the one determined from the iSCAT signal loss of the single nanocube measurements (average volume loss of 33%; Supplementary Figure 24). The fact that the nanocubes retain their cubic shape suggests that decomposition occurs layer-by-layer, consistent with previous reports.[36,37]

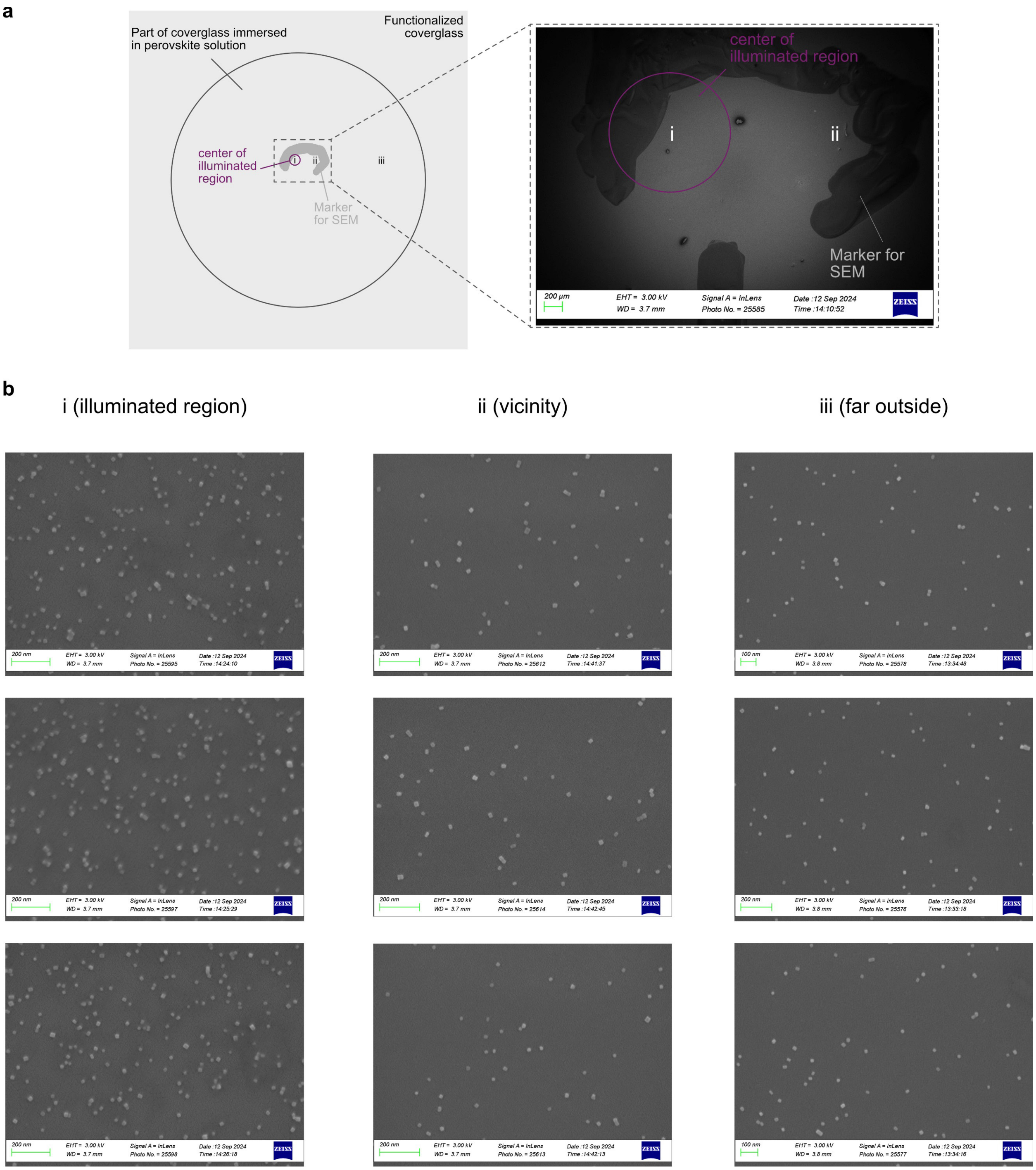

Supplementary Figure 21. a Overview of the sample used in SEM and XPS measurements. A marker was applied to the top of the coverglass for quick localization of the illuminated spot during SEM imaging. The inset shows an SEM image of the region near the illuminated spot. **b** SEM images at higher magnifications of different sample regions. i, directly at the illuminated spot; ii, approximately one millimeter away; iii, a pristine area far from the spot. The images show varying densities of surface coverage by the cubes. Closer to the illumination center, more cubes attached to the surface due to a light-induced effect from the 450 nm laser, a phenomenon frequently observed in experiments. SEM measurements were performed after XPS measurements on the same sample.

b) XPS

To understand how light illumination influences the perovskite composition, we performed XPS measurements over an area containing both a pristine and the illuminated region (see also Supplementary Figure 21a). A marker applied to the underside of the coverglass enabled quick localization of the illuminated region using the camera in the XPS system.

In the illuminated area, a pronounced peak emerged at 136 eV,[38] corresponding to metallic lead (Pb^0) (Supplementary Figure 22a). High-resolution (HR) Pb 4f spectra taken outside (Supplementary Figure 22b) and inside the illumination spot (Supplementary Figure 22c) confirmed the formation of metallic lead (Pb^0) exclusively under light illumination (Pb 4f: 136.3 and 141.2 eV for 7/2 and 5/2, respectively). Additionally, the peak corresponding to Pb^{2+} species (Pb 4f: 138.5 ± 0.1 and 143.4 ± 0.1 eV for 7/2 and 5/2, respectively)[39,40] increased by more than two-fold, consistent with the increase in perovskite material observed at the illumination spot via SEM (Supplementary Figure 21b). An additional peak was observed around 141 ± 0.5 eV (Pb 4f 7/2), exhibiting a similar intensity increase as the Pb^{2+} peak. We attribute this peak to a satellite peak related to the particular environment due to the 3-isocyanatopropyltriethoxysilane functionalization, which facilitates perovskite adsorption.

The peaks observed in the HR Cs 3d and Br 3d spectra (Supplementary Figure 22d, e) were assigned to the perovskite phase (Cs 3d: 724.6 and 738.6 eV for 5/2 and 3/2, respectively; Br 3d: 68.2 and 69.2 eV for 5/2 and 3/2, respectively).[39] There are no significant changes observed upon illumination besides the general increase in signal due to an increased amount of perovskite material. The region in the Br 3d spectra between 68.9 and 70 eV may indicate a signal corresponding to $PbBr_2$. However, the coverglass functionalization increased the contributions of C and O species, diminishing the already weak signal from the single cubes at low surface coverage and limited the resolution of the XPS measurements for the Br and Cs species (< 4% of the XPS signal corresponds to Pb, Cs and Br) as well as the determination of the atomic ratios (considering also the 8-times lower sensitivity factor for Br compared to Pb).

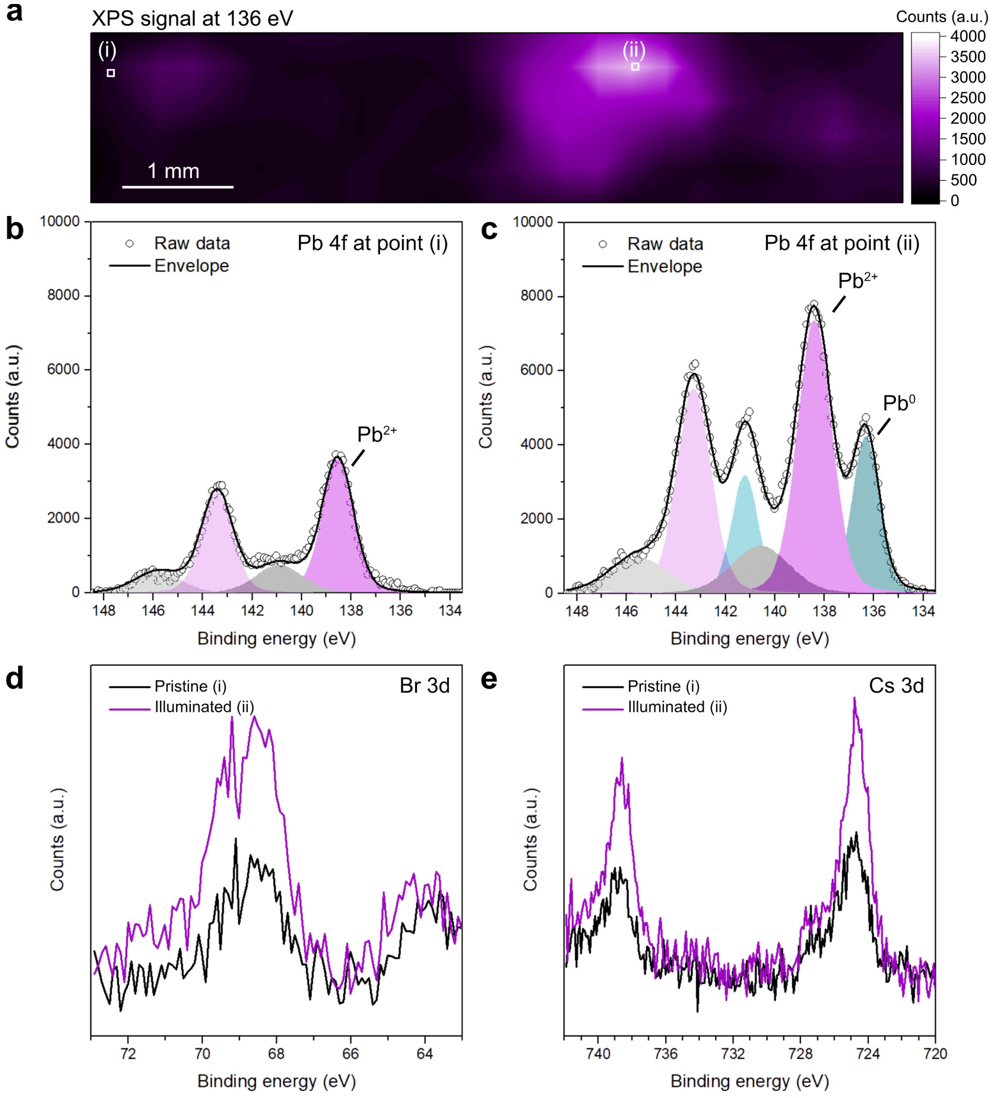

Supplementary Figure 22. Elemental composition evolution of the perovskite due to light illumination. **a** 2D map of the area of interest, coinciding with the illuminated spot. The signal corresponds to the XPS signal count at 136 eV (metallic lead) (map is corrected by background signal counts taken at 133 eV). **b,c** High resolution spectra of Pb 4f for points (i) and (ii) in (a), respectively, revealing the increase in Pb^{2+} and the rise of Pb^0 at the center of the illuminated spot. **d,e** High resolution spectra of Br 3d (d) and Cs 3d (e) acquired in the pristine and the illuminated area, corresponding to the point (i) and (ii) in (a), respectively.

Supplementary Figure 23a shows the evolution of the Pb 4f HR-spectra along a line scan in the x-axis direction. For the line scan passing through the illuminated area (Supplementary Figure 23b), an increase in the Pb^0 species is observed. In contrast, the line scan taken slightly below the illuminated area (Supplementary Figure 23c) shows no signal for Pb^0. No significant changes are detected in the Br and Cs species between the two line scans, apart from a general increase in signal due to the presence of more perovskite material (Supplementary Figures 23d, e).

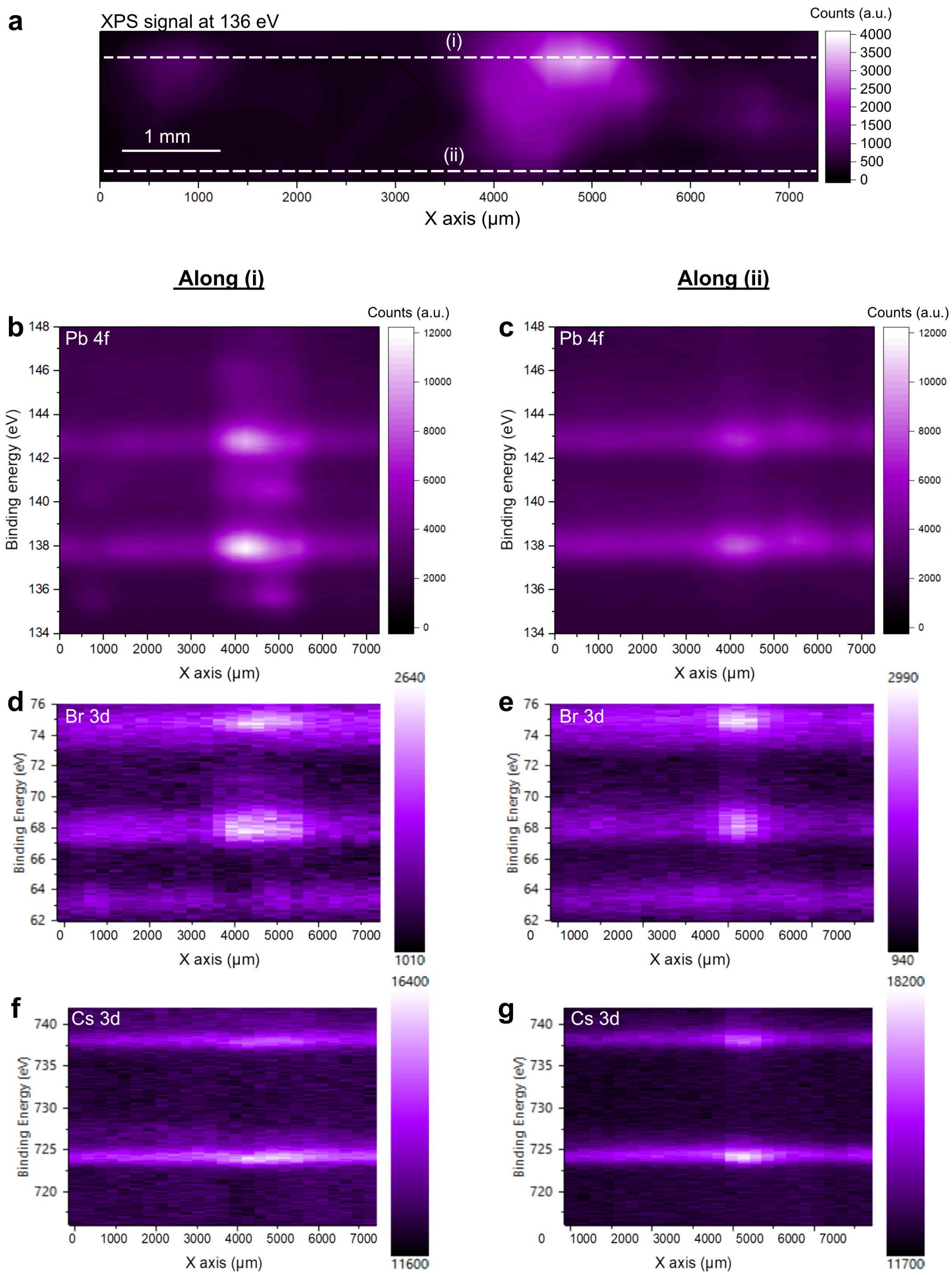

Supplementary Figure 23. Elemental composition evolution of the perovskite due to light illumination. **a** 2D map of the area of interest, coinciding with the illuminated spot. The signal corresponds to the XPS signal count at 136 eV (metallic lead) (map is corrected by background signal counts taken at 133 eV). **b,c** Two-dimensional heat maps of the Pb 4f XPS signal for the line scans (i) and (ii) in (a), respectively. The x-axis depicts the spatial coordinate (in micrometers) along the line scans, while the y-axis represents the binding energy (eV) of the Pb 4f region. Color intensity indicates the magnitude of the measured signal (counts, a.u.), with lighter regions signifying higher signal intensity. **d, e** Two-dimensional heat maps of the Br 3d XPS signal for the line scans (i) and (ii) in (a), respectively. **f, g** Two-dimensional heat maps of the Cs 3d XPS signal for the line scans (i) and (ii) in (a), respectively.

Section 13. Lumerical Simulations – 450 nm and degradation study

a) iSCAT contrast-to-volume formula at 450 nm illumination

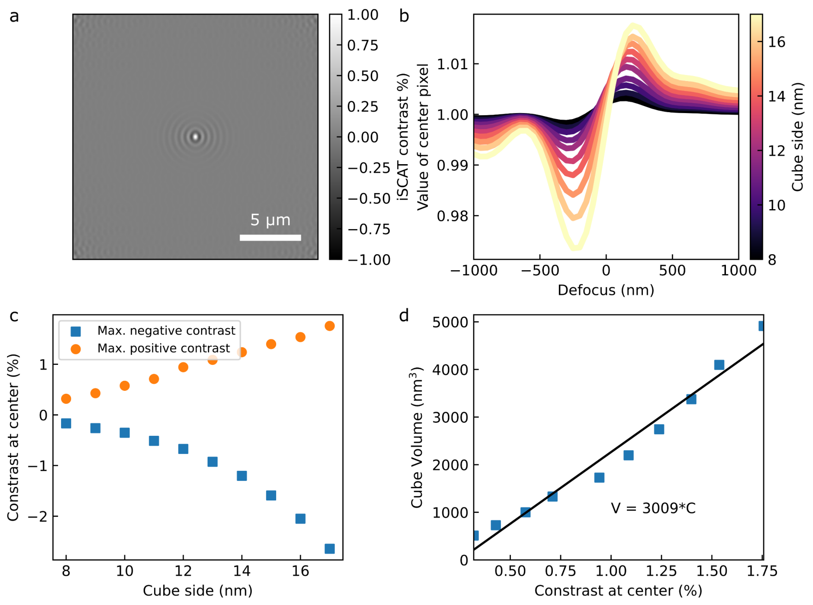

Supplementary Figure 24. Lumerical simulations at λ = 450 nm in hexane (n = 1.375[25]) for $CsPbBr_3$ cubes[41] to obtain the contrast to size conversion formula at 450 nm illumination. **a** Simulated image at maximum positive contrast for a L = 13 nm cube. **b** Value of center pixel for different cube sizes vs. the defocus position, showing a minimum and maximum that do not correspond to the focal position because of the Gouy phase shift. **c** Contrast at image center vs. cube side length plot at the defocus positions yielding the maximum positive and negative contrast values. **d** Cube volume vs. iSCAT contrast at image center for the maximum positive defocus. The linear fit gives the contrast-to-volume formula.

b) Simulations on impact of a loss of absorption properties or a transformation into $PbCO_3$ on the iSCAT contrast

We simulated the effect of perovskite conversion into other materials (e.g. $PbCO_3$) on the iSCAT signal. Our results suggest that the primary influence is indeed the size of the scattering object and not the specific material (Supplementary Figure 25, Supplementary Figure 26), consistent with literature reports of size reduction.[36,37]

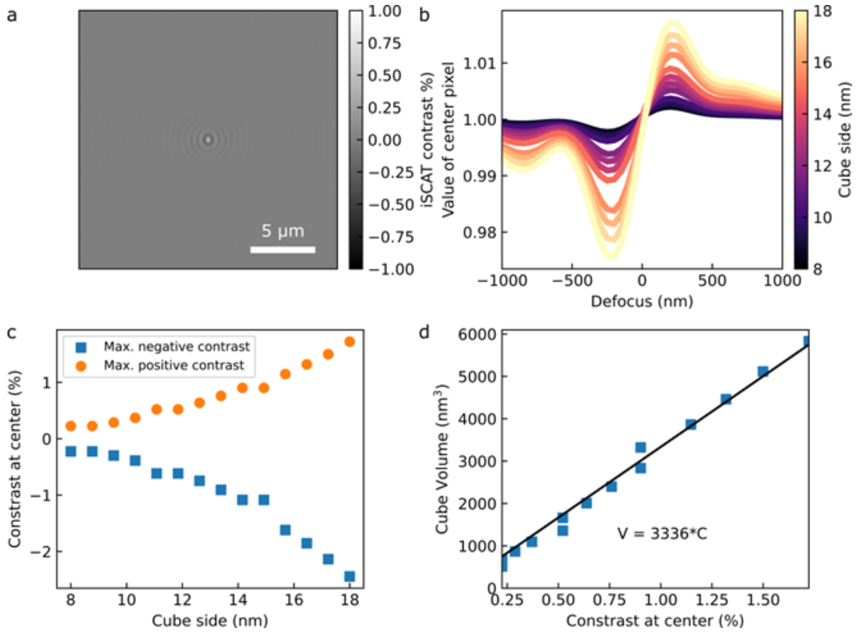

Supplementary Figure 25. Lumerical simulation at $\lambda = 450$ nm to assess the impact of a refractive index change due to a transformation of perovskite to $PbCO_3$[42,43] ($n = 1.8$) on the iSCAT contrast. The volume to contrast relation obtained in this case indicates that conversion to $PbCO_3$ would only result in a 10% contrast reduction, indicating the prominent role of size reduction.

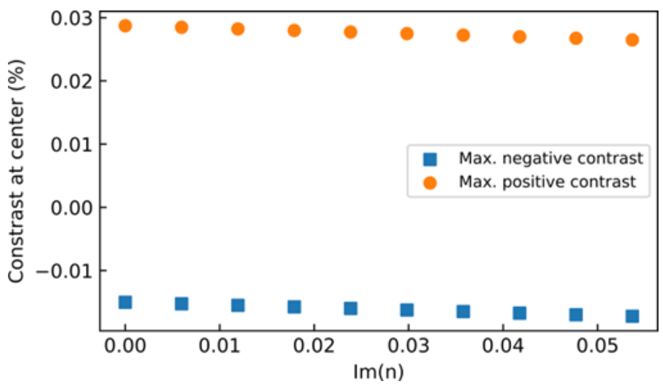

Supplementary Figure 26. Lumerical simulation to assess the impact of the loss of absorption properties of perovskite on the iSCAT contrast. For this we change the imaginary part of dielectric function of the $CsPbBr_3$ perovskite material. Contrast at image center vs. different imaginary parts for a 21 nm cube, showing that the contrast changes only marginally.

Section 14. Volume dependence of degradation

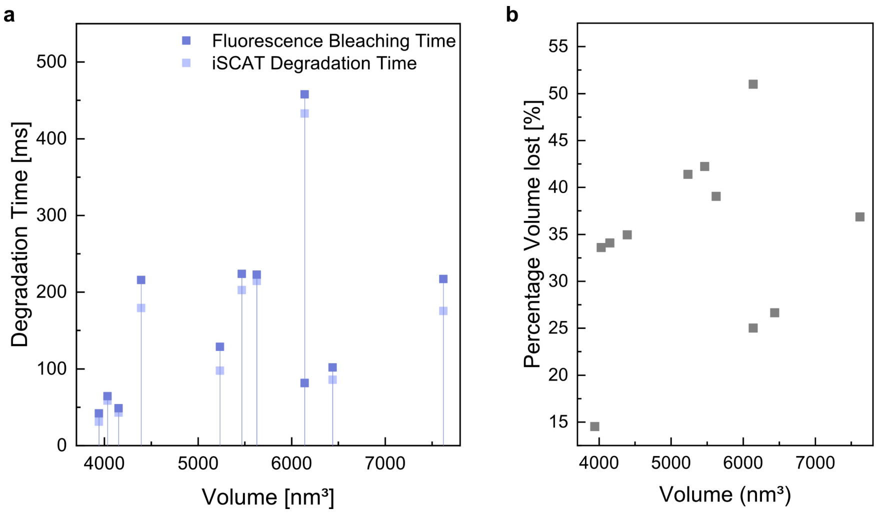

Supplementary Figure 27. Dependence of the degradation process on the initial volume of the cube. **a** Plot of iSCAT and PL degradation time versus initial cube volume. The degradation times for iSCAT and PL are nearly identical, with no apparent trend between degradation time and initial cube volume. **b** Plot of percentage of volume lost versus initial cube volume. There is no clear trend observed.

Section 15. Minimal degradation model

We propose here a minimal model that can reproduce the key features of the degradation experiments on single nanocubes. We aim to capture the surprising experimental trend of decreasing relative amount of lost volume for increasing laser power (Fig. 4e). The fact that the degradation process stops without completely destroying the nanocubes, points to the presence of competing mechanisms: an effect that removes part of the crystal with rate k_r and one that protects part of the crystal from being degraded with rate k_p. This model is consistent with our picture of accumulation of Pb^0 at the nanoparticles surface that halts the degradation process. We can then write a system of coupled differential equations in the particle volume $V(t)$ and the amount of Pb^0 (represented by $C(t)$) that prevents degradation. $C(t)$ varies from 0 (no Pb^0) to 1 (enough Pb^0 to stop the degradation):

$$\frac{dV}{dt} = -k_r P V_0 \big(1 - C(t)\big); \quad \frac{dC}{dt} = k_p P^\gamma (1 - C(t))$$

Here, P is the laser intensity. In this model, the degradation rate depends on the laser intensity P, the initial particle volume V_0, the amount of Pb^0 $C(t)$ and a characteristic constant k_r. While realistically the degradation rate should reduce as the particle size shrinks because of reduced absorption, we use here V_0 to keep the solution simple. The expression for $C(t)$ is:

$$C(t) = 1 - e^{-k_p P^\gamma t}$$

Meaning that for t going to infinity we expect a sufficient amount of Pb^0 to accumulate so that $C = 1$ and consequently the volume dissipation stops. We also assume there is no Pb^0 before the beginning of the degradation process so $C_0 = 0$. In general, the power dependency of the coverage dynamics can be nonlinear in the laser power P; we express this through the coefficient γ and will see that $\gamma \neq 1$ is necessary to yield a power-dependent residual volume, which we observe in the experiments. By substituting $C(t)$ into the differential equation for the volume and integrating, we get the solution:

$$V(t) = V_0 \left[1 - \frac{k_r}{k_p} P^{1-\gamma} \big(1 - e^{-k_p P^\gamma t}\big)\right]$$

The final remaining volume is then

$$V(t = \infty) = V_0 \left(1 - \frac{k_r}{k_p} P^{1-\gamma}\right)$$

With the relative amount of volume removed from the particle being $\frac{k_r}{k_p} P^{1-\gamma}$. We can also define a characteristic decay time $\tau = 1/k_p P^\gamma$ for the volume dissipation, which is a decreasing function of laser power, consistent with experimental observations (Fig. 4e of the manuscript). We can now analyze the results of the model depending on the value of the parameter γ governing the scaling behavior of the Pb^0 formation as a function of laser power.

- If $\gamma = 1$: the formation of Pb^0 is linear in the laser power. The relative amount of volume removed is k_r/k_p and is power-independent, which is not consistent with our experimental observations.
- If $\gamma < 1$: the protective Pb^0 rate has a sublinear power scaling. We expect in this case the Pb^0 formation to be less efficient at higher power. The exponent in the lost volume expression $\frac{k_r}{k_p}P^{1-\gamma}$ is positive, leading to an increase in the removed volume at higher powers, which is the opposite of what we observe experimentally.
- If $\gamma > 1$: the Pb^0 formation rate increases more than linearly with the power. Therefore, for higher power we expect the Pb formation to more effectively quench the degradation process. The power exponent in the lost volume expression $\frac{k_r}{k_p}P^{1-\gamma}$ is negative, leading to a diminishing amount of particle degradation with increasing power, consistent with our experimental observations.

Moreover, the model is also consistent with two additional observations we make from our experiments: both the characteristic dissipation time-scale $\tau = 1/k_pP^\gamma$ and the relative amount of mass lost by the particle $\frac{k_r}{k_p}P^{1-\gamma}$ show no dependence on the initial cube size (Supplementary Figure 27).

References

1. Taylor, R. W. & Sandoghdar, V. Interferometric Scattering Microscopy: Seeing Single Nanoparticles and Molecules via Rayleigh Scattering. *Nano Lett.* **19**, 4827 (2019).

2. Gruber, C. G., Frey, L., Guntermann, R., Medina, D. D. & Cortés, E. Early stages of covalent organic framework formation imaged in operando. *Nature* **630**, 872–877 (2024).

3. Hwang, J. & Moerner, W. E. Interferometry of a single nanoparticle using the Gouy phase of a focused laser beam. *Opt. Commun.* **280**, 487 (2007).

4. Taylor, R. W. & Sandoghdar, V. Interferometric Scattering (iSCAT) Microscopy and Related Techniques. in *Label-Free Super-Resolution Microscopy.* (ed. Astratov, V.) 25–65 (Springer, Cham., 2019). doi:10.1007/978-3-030-21722-8_2.

5. Ortega-Arroyo, J. & Kukura, P. Interferometric scattering microscopy (iSCAT): new frontiers in ultrafast and ultrasensitive optical microscopy. *Phys. Chem. Chem. Phys.* **14**, 15625 (2012).

6. Orfield, N. J. *et al.* Quantum Yield Heterogeneity among Single Nonblinking Quantum Dots Revealed by Atomic Structure-Quantum Optics Correlation. *ACS Nano* **10**, 1960–1968 (2016).

7. Henke, N. A. *et al.* Synthesizer: Chemistry-Aware Machine Learning for Precision Control of Nanocrystal Growth. *Adv. Mater.* **38**, e09472 (2026).

8. Knoll, B. & Keilmann, F. Enhanced dielectric contrast in scattering-type scanning near-field optical microscopy. *Opt. Commun.* **182**, 321–328 (2000).

9. Young, G. *et al.* Quantitative mass imaging of single biological macromolecules. *Science* **360**, 423 (2018).

10. Kowal, M. D. *et al.* Electrophoretic Deposition Interferometric Scattering Mass Photometry. *ACS Nano* **18**, 10388–10396 (2024).

11. Kashkanova, A. D., Blessing, M., Gemeinhardt, A., Soulat, D. & Sandoghdar, V. Precision size and refractive index analysis of weakly scattering nanoparticles in polydispersions. *Nat. Methods* **19**, 586–593 (2022).

12. Kratochvíl, J. *et al.* Best practice mass photometry: a guide to optimal single-molecule mass measurement. *Nat. Protoc.* 1–25 (2025) doi:10.1038/s41596-025-01255-4.

13. Würth, C., Grabolle, M., Pauli, J., Spieles, M. & Resch-Genger, U. Relative and absolute determination of fluorescence quantum yields of transparent samples. *Nat Protoc* **8**, 1535–1550 (2013).

14. Dieperink, M., Skorikov, A., Claes, N., Bals, S. & Albrecht, W. Considerations for electromagnetic simulations for a quantitative correlation of optical spectroscopy and electron tomography of plasmonic nanoparticles. *Nanophotonics* **13**, 4647–4665 (2024).

15. Derkachova, A., Kolwas, K. & Demchenko, I. Dielectric Function for Gold in Plasmonics Applications: Size Dependence of Plasmon Resonance Frequencies and Damping Rates for Nanospheres. *Plasmonics* **11**, 941–951 (2016).

16. Stoller, P., Jacobsen, V. & Sandoghdar, V. Measurement of the complex dielectric constant of a single gold nanoparticle. *Opt. Lett.* **31**, 2474 (2006).

17. Mader, M., Benedikter, J., Husel, L., Hänsch, T. W. & Hunger, D. Quantitative Determination of the Complex Polarizability of Individual Nanoparticles by Scanning Cavity Microscopy. *ACS Photonics* **9**, 466–473 (2022).

18. Griffiths, J. T. *et al.* Effect of Size on the Luminescent Efficiency of Perovskite Nanocrystals. *Acs Appl Energy Mater* **2**, 6998–7004 (2019).

19. Kukura, P., Celebrano, M., Renn, A. & Sandoghdar, V. Imaging a Single Quantum Dot When It Is Dark. *Nano Lett* **9**, 926–929 (2009).

20. Ortiz, F. A. R. *et al.* The Anisotropic Complex Dielectric Function of CsPbBr3 Perovskite Nanorods Obtained via an Iterative Matrix Inversion Method. *J. Phys. Chem. C* **127**, 14812–14821 (2023).

21. Singldinger, A. *et al.* Design Rules for Perovskite Nanocrystals: Volume-Governed Absorption Versus Shape-Controlled Auger Recombination. *Adv. Opt. Mater.* (2025) doi:10.1002/adom.202501137.

22. Tian, Y., Halle, J., Wojdyr, M., Sahoo, D. & Scheblykin, I. G. Quantitative measurement of fluorescence brightness of single molecules. *Methods Appl. Fluores.* **2**, 035003 (2014).

23. Photons, Electrons, and Gray Levels. https://www.photometrics.com/learn/white-papers/photons-electron-and-gray-levels (2020).

24. Allan, D. B., Caswell, T., Keim, N. C., van-der-Wel, C. M. & Verweij, R. W. Trackpy. (2021) doi:10.5281/zenodo.12708864.

25. Polyanskiy, M. N. Refractiveindex.info database of optical constants. *Sci. Data* **11**, 94 (2024).

26. Melhuish, W. H. Quantum efficiencies of fluorescence of organic substances: Effect of solvent and concentration of the fluorescent solute 1. *J. Phys. Chem.* **65**, 229–235 (1961).

27. Yang, Z.-C. *et al.* Intrinsically fluorescent carbon dots with tunable emission derived from hydrothermal treatment of glucose in the presence of monopotassium phosphate. *Chem. Commun.* **47**, 11615–11617 (2011).

28. Mello, J. C. de, Wittmann, H. F. & Friend, R. H. An improved experimental determination of external photoluminescence quantum efficiency. *Adv. Mater.* **9**, 230–232 (1997).

29. Omagari, S., Hirao, E. & Vacha, M. Luminescence Variability of CsPbBr3 Nanocrystals in Single-Particle Emitter Applications. *ACS Appl. Nano Mater.* (2025) doi:10.1021/acsanm.5c03524.

30. Gallagher, S. *et al.* Ligand Equilibrium Influences Photoluminescence Blinking in CsPbBr3: A Change Point Analysis of Widefield Imaging Data. *ACS Nano* **18**, 19208–19219 (2024).

31. Takagi, T., Omagari, S. & Vacha, M. Suppression of blinking in single CsPbBr 3 perovskite nanocrystals through surface ligand exchange. *Phys. Chem. Chem. Phys.* **25**, 19004–19012 (2023).

32. Chouhan, L. *et al.* Real-Time Blinking Suppression of Perovskite Quantum Dots by Halide Vacancy Filling. *ACS Nano* **15**, 2831–2838 (2021).

33. Matsuzaki, K., Liu, H.-W., Götzinger, S. & Sandoghdar, V. On Quantum Efficiency Measurements and Plasmonic Antennas. *ACS Photonics* **8**, 1508–1521 (2021).

34. Lichtenegger, M. F. *et al.* Electron–Hole Binding Governs Carrier Transport in Halide Perovskite Nanocrystal Thin Films. *ACS Nano* **16**, 6317–6324 (2022).

35. Gélvez-Rueda, M. C. *et al.* Interconversion between Free Charges and Bound Excitons in 2D Hybrid Lead Halide Perovskites. *J. Phys. Chem. C* **121**, 26566–26574 (2017).

36. Darmawan, Y. A., Yamauchi, M. & Masuo, S. In Situ Observation of a Photodegradation-Induced Blueshift in Perovskite Nanocrystals Using Single-Particle Spectroscopy Combined with Atomic Force Microscopy. *J. Phys. Chem. C.* **124**, 18770–18776 (2020).

37. Yuan, G. *et al.* The Degradation and Blinking of Single CsPbI3 Perovskite Quantum Dots. *J. Phys. Chem. C* **122**, 13407–13415 (2018).

38. Ali, A. *et al.* The Electronic Impact of Light-Induced Degradation in CsPbBr3 Perovskite Nanocrystals at Gold Interfaces. *J. Phys. Chem. Lett.* **15**, 3721–3727 (2024).

39. Kim, H.-R. *et al.* Cesium Lead Bromide (CsPbBr3) Perovskite Quantum Dot-Based Photosensor for Chemiluminescence Immunoassays. *ACS Appl. Mater. Interfaces* **13**, 29392–29405 (2021).

40. Shi, T. *et al.* Scalable synthesis of ultrastable lead halide perovskite-zeolite composites via a chemical vapor method in air. *NPG Asia Mater.* **14**, 87 (2022).

41. Whitcher, T. J. *et al.* Dual phases of crystalline and electronic structures in the nanocrystalline perovskite CsPbBr3. *NPG Asia Mater.* **11**, 70 (2019).

42. Huang, S. *et al.* Morphology Evolution and Degradation of CsPbBr3 Nanocrystals under Blue Light-Emitting Diode Illumination. *ACS Appl. Mater. Interfaces* **9**, 7249–7258 (2017).

43. Li, J. *et al.* Ultraviolet light induced degradation of luminescence in CsPbBr3 perovskite nanocrystals. *Mater. Res. Bull.* **102**, 86–91 (2018).
